# Cytotoxic Compounds Derived from Marine Sponges. A Review (2010–2012)

**DOI:** 10.3390/molecules22020208

**Published:** 2017-01-28

**Authors:** Roberto Mioso, Francisco J. Toledo Marante, Ranilson de Souza Bezerra, Flávio Valadares Pereira Borges, Bárbara V. de Oliveira Santos, Irma Herrera Bravo de Laguna

**Affiliations:** 1 Laboratory of Enzymology – LABENZ, Department of Biochemistry, Federal University of Pernambuco, Recife 50670-901, Pernambuco, Brazil; ransoube@uol.com.br; 2 Department of Chemistry, University of Las Palmas de Gran Canaria, Las Palmas de Gran Canaria 35017, Spain; ftoledo@dqui.ulpgc.es; 3 Post-Graduation Program in Natural Products and Synthetic Bioactives, Federal University of Paraíba, João Pessoa 58051-970, Paraíba, Brazil; flavinhovb@hotmail.com; 4 Post-Graduation Program in Development and Technological Innovation in Medicines, Department of Pharmaceutical Sciences, Federal University of Paraiba, João Pessoa 58051-900, Paraíba, Brazil; 5 Department of Biology, University of Las Palmas de Gran Canaria, Las Palmas de Gran Canaria 35017, Spain

**Keywords:** porifera, marine sponges, pharmacology, bioactive molecules, cytotoxicity, cancer cell lines

## Abstract

This extensive review covers research published between 2010 and 2012 regarding new compounds derived from marine sponges, including 62 species from 60 genera belonging to 33 families and 13 orders of the Demospongia class (Porifera). The emphasis is on the cytotoxic activity that bioactive metabolites from sponges may have on cancer cell lines. At least 197 novel chemical structures from 337 compounds isolated have been found to support this work. Details on the source and taxonomy of the sponges, their geographical occurrence, and a range of chemical structures are presented. The compounds discovered from the reviewed marine sponges fall into mainly four chemical classes: terpenoids (41.9%), alkaloids (26.2%), macrolides (8.9%) and peptides (6.3%) which, along with polyketides, sterols, and others show a range of biological activities. The key sponge orders studied in the reviewed research were Dictyoceratida, Haplosclerida, Tetractinellida, Poecilosclerida, and Agelasida. *Petrosia*, *Haliclona* (Haplosclerida), *Rhabdastrella* (Tetractinellida), *Coscinoderma* and *Hyppospongia* (Dictyioceratida), were found to be the most promising genera because of their capacity for producing new bioactive compounds. Several of the new compounds and their synthetic analogues have shown in vitro cytotoxic and pro-apoptotic activities against various tumor/cancer cell lines, and some of them will undergo further in vivo evaluation.

## 1. Introduction

Sponges (Porifera), the phylogenetically oldest metazoan phylum still in existence today, have been established as being an exceptionally valuable source of new marine natural products. As a consequence of their evolutionary development—mainly regarding their sessile mode of life, their porous and multicelled colonial form—sponges produce a range of secondary metabolites, mostly alkaloids as good toxicants, that play an important role as allomones and protect them against predators and fouling organisms [1,2]. Therefore, it is clear that these apparently defenseless organisms were endowed by evolution with strong allelopathic factors and, by logical deduction, could be authentic bio factories of new drugs of natural origin.

Besides elements of alkaloidal nature, sponges also biosynthesize other interesting classes of natural products such as terpenoids, glycosides, phenols, phenazines, polyketides, fatty acids, peptides, amino acid analogues, nucleosides, porphyrins, aliphatic cyclic peroxides and sterols [3,4,5], whose chemical structures display highly complex frameworks and are factually relevant per se.

Once isolated in a laboratory, these substances can display strong and often highly specific biological activities such as antibacterial, antiviral, antifungal, anti-prion, antimalarial, anti-inflammatory and immune or neuro-suppressive qualities [6,7,8,9]. They exhibit, moreover, pronounced cytotoxic activity towards certain types of malignant cell lines, which make them potential drug targets for the treatment of multifactorial diseases such as cancer [10,11]. Additionally, when exposed to a cytotoxic compound, healthy living cells can also be induced to undergo either necrosis (accidental cell death) or apoptosis (programmed cell death).

DNA damage and consequent apoptosis are leading cytotoxic mechanisms of several anticancer agents [12]; therefore, certain assays measure DNA damage and apoptosis. In fact, cell cytotoxicity refers to the ability of certain chemicals or mediator cells to damage or destroy living cells which are reproducing. Therefore, the measuring of cell cytotoxicity is quite indispensable for the development of therapeutic anti-cancer drugs [13].

Cell cytotoxicity can be measured in a number of different ways. Assessing cell viability through the use of vital dyes (formazan dyes), protease biomarkers or by measuring ATP content are some of the most commonly used methods [14,15]. The colorimetric method, known as MTT in vitro cell viability assay—developed by Mossman [16]—and its derivatives (MTS/PMS), are still the most economic, versatile, and widespread assay types for evaluating preliminary anticancer activity in both synthetic derivatives and natural products [17]. Therefore, endeavoring to understand the mechanisms of action involved in cytotoxicity can certainly give researchers more in-depth knowledge into the biological processes governing the growth, proliferation and death of cells [18].

By far, molecules from sponges with antineoplastic properties—inhibiting or preventing the growth and spread of tumors—have great significance for chemistry, pharmacology and medicine, and constitute still a big unknown to researchers [19]. The characterization of these bioactive compounds may encourage synthetic proposal and other biotechnological procedures for the large-scale production of these challenging structures [20,21,22,23], while these renewable approaches may also contribute to preserve the wild stocks of sponges as natural repositories of these molecules.

Cancer is documented as a complex group of diseases caused by interactions of multiple factors such as genetic susceptibility, environmental and lifestyle influences, infectious agents and ageing [24], for which traditional chemotherapies and targeted anticancer treatments exert their effects by direct cytotoxicity or tumor growth inhibition. Nevertheless, the curative effects of these chemotherapeutic drugs are not always good enough and are often associated with numerous side-effects. Consequently, the search for highly efficient anticancer therapies remains the biggest challenge in medicine [25].

Literature records reveal that many natural products isolated from marine sponges have displayed a wide range of antineoplastic properties. Nowadays, more than five thousand compounds have been isolated from these metazoans whereas over a hundred new molecules are reported each year [8,11,26,27,28,29,30], and most interesting: a large part of them exhibit low micromolar cytotoxicity towards a range of human cancer cell lines.

Therefore, the most conventional method for identifying possible chemotherapeutic agents appears to be the mass screening of natural products for their cytotoxic activities allied with an effective set of dereplication strategies in order to increase the efficiency of the lead compounds discovery process [30]. In vivo and in vitro chemopreventive/anticancer research involves the evaluation of antiproliferative, antiangiogenic and apoptosis induction potentials of the isolated compounds [12,31,32,33].

Cytotoxic assays represent a bottleneck for any potential bioactives before they undergo preclinical and clinical trials, in particular those compounds tested against malignant cell lines. The use of biological models systems to access indirect in vivo toxicity (e.g., *Artemia* nauplii and echinoderm eggs/embryos assays) still constitutes a useful tool for preliminary assess to lethality produced by marine natural products [34,35], while a more sophisticated approach (secondary screen) corresponds to the access to in vitro cytotoxicity carried out on animals (e.g. murine tumor lines) or human cancer cells lines [13,36]. However, and as general rule, it is important to remember that toxic components denote pharmacological interest always at low-micromolar concentration, defined as an IC_50_ value of ≤10 µM (or 4–5 µg·mL^−1^) [37], below which they would appear as possible candidates for additional mechanism-of-action studies.

Pancreatic cancer, for example, is considered one of the most malignant tumors mainly due to its aggressive metastasis spreading and its high resistance to apoptosis. Moreover, Manzamine A (HB-071) is a known β-carboline alkaloid biosynthesized by diverse marine sponges that has been tested on Panc-1 ATCC^®^ CRL-1469^™^ (*Homo sapiens* pancreas/duct epithelial) cancer cell lines. Thus, its potent cytotoxic effect has been assayed in vitro against this disease, at low micromolar dosage (5 and 10 µM), showing to be an effective antitumor lead compound once it has restored sensitivity to apoptosis [38].

Given the importance of their cytotoxic effects, numerous natural products isolated from marine-sponges and their analogues and derivatives, have been synthesized to date [39,40,41,42]. These have proven to be—these and other bioassays—an important tool for developing potentially useful anticancer agents that are mainly naturally resistant to proapoptotic stimuli such as glioblastomas, melanomas, non-small-cell-lung cancers and metastatic cancers [43,44]. Thus, and beyond the significance of these chemical structures—and additionally, a number of synthetic studies have also been included in this work.

### 1.1. Taxonomy of Marine Sponges

The phylogeny of marine sponges has been established exclusively with structural and morphological characteristics whose results were withheld in *Systema Porifera* (*SP*), a reference guide to the classification of sponges edited by Hooper and van Soest [45]. However, the implementation of molecular techniques directed to the phylogenetic analyses of different genes has progressively readjusted the topology of part of the Porifera tree.

Although the *SP* can be considered out of date, it has developed the systematics of the phylum until recently, organizing the available knowledge regarding these multifaceted organisms and serving as an important supporting tool to the spongologists and natural product researchers. Notwithstanding, the World Porifera Database (WPD) [46], which is the web’s largest world database of all recent sponges ever described, is complementary to the aforementioned *SP* and incorporates the latest and future amendments of the sponge classification, including the recently proposed revision by Morrow and Cárdenas [47].

Thus, in order to improve reading comprehension and provide easy access to the scientific information, the structure of this article has been organized in alphabetical sequence, particularly, based on the phylogenetic analysis of the sponges whose classification was revised by Morrow and Cárdenas [47]. Nevertheless, this work should be understood first as an element of guidance, from which the readers can deepen their knowledge with simply accessing a specific database, and looking for a specific topic of their interest.

### 1.2. Research and Methodology

This review provides concise information about the chemical diversity found in the Demospongiae class of sponges particularly about secondary metabolites, where a deep bibliographic search was done across the SciFinder, ScienceDirect and PubMed databases, using the keywords “sponge” on the one hand and “activity” on the other. Also, a supplementary bibliographic search was carried across the SciFinder, to seek out biological activities and synthetic proposals from metabolites described for each genus of sponges that were named in the review.

During the review preparation, several searches were made to locate digital records of theses and dissertations at various international institutions. In all assessments, a few restrictive terms were used: “porifera” or “sponge” and “activity or activities”. After obtaining the results, we excluded duplicates as well as references to other “sponges” (e.g., plant, surgical or metal), and records on paleontology and sedimentology.

Publications on taxonomic descriptions were also used to establish the list of species, which was organized in some measure according to the taxonomic structure adopted in *Systema Porifera* [45], which was revised by Morrow and Cárdenas [47]; and finally contrasted with the information offered in WPD, available online at http://www.marinespecies.org/porifera [46].

On the other hand, and for a better understanding of this work, it is assumed that all chemical structures mentioned in the manuscript were characterized by detailed spectroscopic analysis, and comparison of their spectroscopic data with those of related model compounds reported in the literature, while the absolute stereochemistry of certain structures was defined by analysis of the coupling constants and optical rotation, among other techniques.

The emphasis of this proposal rests on the pharmacological activities, especially regarding the cytotoxic activity, where several of these metabolites were evaluated against a broad range of malignant cell lines obtained in different cell banks across the world. Moreover, some molecular structures were added to illustrate the manuscript and as a didactic form to improve the understanding of the work, allowing the reader explore several facets of organic chemistry and biological aspects of these intriguing organisms.

Accordingly, this manuscript was organized taking into consideration a hierarchical taxonomic archetypal. The families were described within each order and the known genera with their correspondingly species (identified or not) were described within each family, along their described structural groups of natural products and cytotoxic properties recorded during the triennium 2010–2012.

## 2. Porifera Involved in the Biosynthesis of Cytotoxic Metabolites

The phylum porifera is classified in three major classes, namely Calcareae (calcareous sponges), Hexactinellidae (glass sponges) and Demospongiae (horny sponges). However, only species belonging to the class Demospongia were considered in this review since this group of Porifera includes almost all the records on natural products—chemical structures and their pharmacological properties—found in the database search constrained to the records between 2010 and 2012.

### 2.1. Class: Demospongiae

The Demospongiae higher taxa classification has been recently revised based on the reevaluation of their morphological characters along with the molecular data which were recompiled from the scientific literature over the last decade [47]. As a result, the spongologists have proposed the use of 22 orders termed Agelasida, Axinellida, Biemnida, Bubarida, Chondrillida, Chondrosiida, Clionaida, Dendroceratida, Desmacellida, Dictyoceratida, Haplosclerida, Merliida, Poecilosclerida, Polymastiida, Scopalinida, Sphaerocladida, Spongillida, Suberitida, Tethyida, Tetractinellida, Trachycladida and Verongiida, which belong to the known subclasses Heteroscleromorpha, Keratosa and Verongimorpha.

#### 2.1.1. Order: Agelasida

The order Agelasida has been preserved from *SP* and involves two families: Agelasidae and Astroscleridae [48,49]. However, during the time interval in which the review was conducted in the database, it has not been detected natural products isolated from sponges belonging to the latter family, the Astroscleridae.

##### Family: Agelasidae

The family Agelasidae formerly was a monotypic family having the genus *Agelas* as its unique representative [49]. However, the taxon was revised and it has received two new genera, *Acanthostylotella* and *Amphynomia*, both initially assigned to the family Raspailiidae (Poecilosclerida) [48]. As per the SciFinder database, this small group of sponges is known to contain terpenoids (diterpenes and alkaloids based on terpenoid skeletons), free steroids, alkaloids (bromopyrrole and *N*-methyladenine-containing alkaloids, fluorinated agelastatin analogues, pyrrole carboxylic acids- and aminoimidazol- derivatives), C_11_N_5_ diketopiperazines, α-linked galactosylceramides (cerebrosides), glycolipids, carotenoids, fatty acids, bisuracil analogs and other types of chemical components. These metabolites display several biological activities including cytotoxic, antitumor (anticancer), antiangiogenic, as an inhibitors of protein tyrosine phosphatase 1B (PTP1B), antimicrobial (antibacterial and antifungal), and antiprotozoal (antileishmanial and trypanocidal) activities.

**Figure 1:**
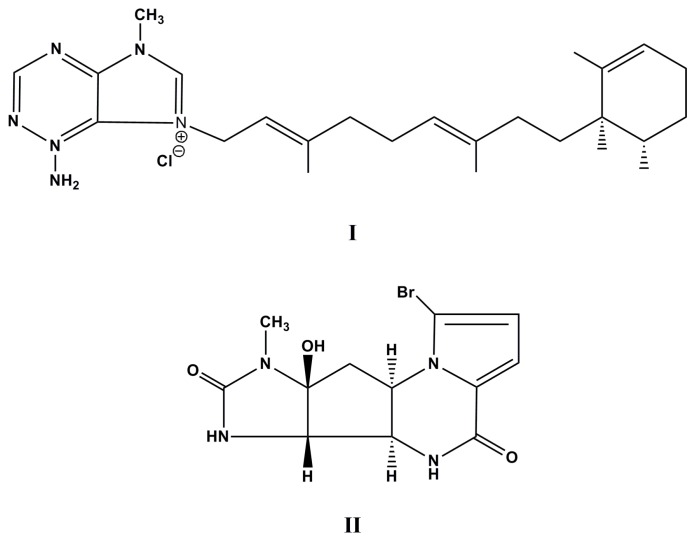
Agelasine F (**I**) and (−)-agelastatin A (**II**).

In the search for new bioactive compounds from the sea, Calcul et al. have collected an unidentified specimen of marine sponge belonging to the genus *Agelas*, and they have isolated four novel 9-*N*-methyladeninium diterpenoids named agelasine M, 2-oxo-agelasine B, gelasine A, and gelasine B accompanied by the known agelasines B and F [49]. According to theses authors, all pure compounds were evaluated for cytotoxicity against Jurkat T-leukemia cells (clone E6-1). Nonetheless, strong cytotoxicity was only observed for agelasine F (**I**, Figure 1) with IC_50_ growth inhibitory concentration value of 3.3 µg·mL^−1^ but not for the new derivative agelasine M. The search for records revealed that a total synthesis of ent-agelasine F had already been achieved, starting from commercially available (*R*)-pulegone [50].

The chemical investigation of the marine sponge *Agelas citrina*, that was collected at Little San Salvador, Bahamas, yielded three new diterpene alkaloids, the hypotaurocyamines named (−)-agelasidines E and F, and an adeninium salt [51]. According with this work, the known agelasine N was also isolated along with six known natural products, agelasines B–E, 2-oxo-agelasine B, and (−)-agelasidine C. (−)-Agelasidine C has exhibited weak cytotoxic activity against human chronic lymphocytic leukemia (CLL) cell lines with IC_50_ value of 10^−3^ M. A total synthesis of agelasidine C had been accomplished by Asao et al. [52].

On the other hand, the butanolic extract from *Agelas dendromorpha* led the isolation of three new pyrrole-2-aminoimidazole (P-2-AI) alkaloids, named agelastatins E and F and benzosceptrin C, together with 10 known metabolites, agelastatins A and D, sceptrin, manzacidin A, tauroacidin A, taurodispacamide A, nortopsentin D, thymine, longamide, and 4,5-dibromopyrrole-2-carboxamide. The pure compounds were screened for cytotoxic activity against the human nasopharyngeal epidermoid carcinoma (KB) cell lines and, except for (−)-agelastatin A (**II**, Figure 1) which has shown 100% activity at 30.0 and 3.0 μM. All other compounds have shown no significant bioactivity at 30.0 µM [53]. In a recent study, natural (−)-agelastatin A has exhibited potent in vitro activity against primary CLL cell lines, and it has disclosed the synthesis of several analogues that were equipotent or exceed the potency of the naturally occurring product [54].

#### 2.1.2. Order: Axinellida

The order Axinellida, not present in *SP*, has been recently resurrected and encompasses the families Axinellidae, Heteroxyidae, Raspailiidae and Stelligeridae [47]. Some bioactive metabolites from species belonging to three families of this order are detailed below.

##### Family: Axinellidae

The Axinellidae family covers the genera *Axinella*, *Dragmacidon*, *Dragmaxia* and *Epipolasis* [47]. Outlined in the SciFinder database, this family of sponges biosynthesizes a wide range of metabolites such as terpenes (triterpenes, bicyclic sesquiterpenes, aromatic sesquiterpenes type alpha-curcumenes, and azulene diterpenes), sterols, alkaloids (acylated taurine and pyridinium derivatives, β-carboline and bromo pyrrole aminoimidazole alkaloids, as well as pyrrole carboxylic acid derivatives), cyclopeptides (C_11_N_5_ diketopiperazines), macrolides of the spirastrellolides family, polyethers, phospholipid fatty acids, glycosphingolipids (cerebrosides), sulfated compounds, nucleosides, and other natural products. These biologically active metabolites show cytotoxic (antitumor), antimicrobial (antibacterial and antifungal), anti-inflammatory and antiprotozoal (antileishmanial) properties.

Thus, spirastrellolides A and B have been isolated as free acids from an undescribed marine sponge of the genus *Epipolasis*. These compounds had been isolated previously from *Spirastrella coccinea* after conversion to the methyl esters. In this work, the cytotoxic activities of spirastrellolides A and B against HeLa cells have shown that the activities of the free acids are comparable to those of the corresponding methyl esters where they had shown citotoxicities, with IC_50_ values in the range of 20.0 to 70.0 nM [55]. Synthetic studies on spirastrellolides had been reported by Smith et al. [40].

On the other hand, one new isonitrile diterpene together with three known ones have been isolated from *Dragmacidon* (*Pseudoaxinella*) *flava*, and were in vitro assayed in human cancer cell lines, using an MTT colorimetric assay and quantitative video microscopy. Two isonitriles have displayed activity for human PC3 prostate apoptosis-sensitive cancer cell lines. These results identify marine diterpene isonitriles as potential lead compounds for anticancer drug discovery. All diterpenes have displayed similar growth inhibitory activities for the human PC3 prostate cancer cell lines, with IC_50_ growth inhibitory values ranging from 1.0 to 7.0 µM for the isonitriles. The IC_50_ growth inhibitory concentrations for two of these isonitriles were 25.0 and 10.0 μM for U373 glioblastoma, 50.0 and 4.0 μM for Hs683 oligodendroglioma, 42.0 and 16.0 μM in A549 NSCLC, 3.0 μM for both compounds for LoVo colon cancer, and 6.0 and 32.0 μM for SKMEL-28 melanoma cell lines, respectively [56].

##### Family: Raspailiidae

Sponges belonging to the family Raspailiidae include species belonging to a wide range of genera such as *Acantheurypon* (Poecilosclerida *incertae sedis*), *Aulospongus*, *Axechina*, *Ceratopsion*, *Cyamon*, *Didiscus*, *Endectyon*, *Ectyoplasia*, *Eurypon*, *Hymeraphia*, *Lithoplocamia*, *Pandaros*, *Ptilocaulis*, *Plocamione*, *Raspaciona*, *Raspailia*, *Reniochalina*, *Rhabdeurypon*, *Sollasella*, *Tethyspira*, *Thrinacophora*, *Trachostylea*, *Trikentrion* and *Waltherarndtia* [47].

As described in the SciFinder database, this group of sponges is known to contain also a wide variety of secondary metabolites such as phenolic sesquiterpenes, diterpenes, triterpenoids, sulfated steroids, steroidal glycosides, acetylated glycolipids, linear and cyclic peptides, phorboxazoles, indole-, guanidine-, and diterpene-alkaloids, acetylenic alcohols, as well as dihydrothiopyranones and diyne-enol-ethers of glycerols. Pharmacological investigation on these secondary metabolites has revealed their bioactive properties: cytotoxic (antitumor), antimicrobial, antiviral (anti-hepatitis B and anti-HIV-1), hemolytic, nematocidal and antiprotozoal (antimalarial) activities.

So, two cytotoxic peptides named yaku’amides A and B were isolated from an undescribed sponge of the genus *Ceratopsion*. Yaku’amides A and B have exhibited potent cell-growth inhibitory activity against P388 murine leukemia cells with IC_50_ values of 14.0 and 4.0 ng·mL^−1^, respectively. In order to analyze the mode of action of yaku’amide A, it was examined its growth inhibitory profile against a panel of 39 human cancer cell lines. Because yaku’amide A exhibits clearly a unique profile as compared to any 38 anticancer drugs, it is suggested that yaku’amide A has a particular mode of action in its growth-inhibitory activity [57]. A synthetic study of yaku’amides A and B has been recently published [58].

#### 2.1.3. Order: Bubarida

The order Bubarida has been erected and encompasses two families, Bubaridae and Dictyonellidae [47]. However, records on natural products isolated from marine sponges belonging to the former family were not detected during the current search on the databases.

##### Family: Dictyonellidae

Marine sponges belonging to the Dictyonellidae family include five valid genera as *Acanthela*, *Axinyssa*, *Cymbastela*, *Dictyonella*, *Lipastrotethya*, *Phakettia* and *Rhaphoxya* [47]. As the SciFinder database also lists, this group of sponges produces several metabolites such as sesquiterpenoid esters, sesquiterpene quinones, polyhydroxi-sterols, polybrominated compounds, diketopiperazines, oxy-polyhalogenated diphenyl ethers, polychlorinated tetra-peptides, polychlorinated pyrrolidinones and other types of compounds. These secondary metabolites exhibit a variety of biological activities such as anti-inflammatory, antiplasmodial, inhibition of the blood coagulation, antimicrobial, antifungal, cytotoxic, antitumor and anti-HIV-I, among others activities.

Therefore, a novel family of functionalized peptide toxins, aculeines (ACUs), was isolated from the marine sponge *Axinyssa aculeata*. The isolation, amino acid sequence, and biological activity of this new group of cytotoxic sponge peptides were described. Also, aculeines A–C have shown moderate cytotoxicity against cultured human cancer cell-lines (MDA-MB-231, A 549, and HT-29), with GI_50_ values of approximately 0.5 µM. No significant difference in potency was observed between the cell lines tested [59].

Nine new triterpene galactosides and aglycons, along with three known compounds from the rare pouoside class, were isolated from *Lipastrotethya* sp. The compounds have exhibited weak to no activity against a K562 human erythroleukemia cell lines with IC_50_ values in the range of 12.5 to 100.0 μM; with IC_50_ value of 13.6 μM for doxorubicin. With two exceptions, the aglycones were far more cytotoxic than the triterpene galactosides [60]. On the other hand, five new nortriterpene glycosides, designated as sarasinosides N–R, along with eight known related compounds, were isolated from another sample of *Lipastrotethya* sp. Several of these new compounds have exhibited moderate to weak cytotoxicity against A549 lung carcinoma and K562 leukemia cell lines [61].

#### 2.1.4. Order: Dictyoceratida

The order Dictyoceratida encompasses four families of marine sponges named Dysideidae, Irciniidae, Spongiidae and Thorectidae [62].

##### Family: Dysideidae

Marine sponges belonging to the Dysideidae family include five valid genera as *Citronia*, *Dysidea*, *Euryspongia*, *Lamellodysidea* and *Pleraplysilla* [63]. Again, as per the SciFinder database, this group of sponges is known to contain sesquiterpenoid esters, sesquiterpene quinones, polyhydroxi-sterols, polybrominated compounds, diketopiperazines, oxy-polyhalogenated diphenyl ethers, tetrapeptides, polychlorinated peptides, polychlorinated pyrrolidinones and other compounds. These metabolites exhibit a variety of biological activities such as cytotoxic (antitumor), anti-HIV-1, antimicrobial (antifungal), anti-inflammatory and antiplasmodial, between other activities.

Thus, the active organic extract of *Dysidea* sp. was subjected to bioassay-guided fractionation to give three new polyoxygenated steroids (dysideasterols F–H), together with two known related compounds. All compounds have exhibited similar cytotoxic effect against human epidermoid carcinoma A431 cells with IC_50_ values of 0.15 to 0.3 µM [64].

Dysidavarones A–D, four new sesquiterpene quinones possessing the unprecedented dysidavarane carbon skeleton, were isolated from the South China Sea sponge *Dysidea avara*. Dysidavarones A and D were evaluated for their cytotoxicity against four human cancer cell lines, cervix (HeLa), lung (A549), breast (MDA231), and hepatoma (QGY7703), by an MTT method, using camptothecin as a positive control. Dysidavarone A has shown a growth inhibitory effect against HeLa cells with an IC_50_ value of 9.9 μM, while dysidavarone D has shown inhibitory effects against the four cell lines with IC_50_ values of 28.8, 21.4, 11.6, and 28.1 μM, respectively [65]. Dysidavarone A was synthesized by Schmalzbauer et al. and by Fukui et al., with 30% overall yield in a longest liner sequence of 13 steps from commercial *o*-vanillin [66,67].

##### Family: Irciniidae

Marine sponges belonging to the family Irciniidae involves three valid genera: *Ircinia*, *Psammocinia* and *Sarcotragus* [68], which contain a rare class of glycinyl lactam sesterterpenes with modulatory properties against alpha-1 and alpha-3 GlyR isoforms [69]. Additionally, as compiled in the SciFinder database, this family produces sterols, sesterterpenes (linear-furano-nor-sesterterpenoids), indole alkaloids, halogenated peptides, polyketides, polyprenyl chromenes, hydroquinone-derivatives and other compounds, which have cytotoxic and anticancer properties.

Chemical investigation of *Sarcotragus spinosulus* led the isolation of a new hydroxylated nonaprenylhydroquinone, along with two known metabolites, hepta and octaprenylhydroquinones. All metabolites were evaluated for their potential antileukemic effect towards the human chronic myelogenous (CML) cell lines K562. It was observed that the hydroxylated nonaprenylhydroquinone and octaprenylhydroquinone have inhibited cell metabolism and cell number with very similar IC_50_ values, around of 10.0 μM. Octaprenylhydroquinone was less efficient that the two former compounds with IC_50_ values of 193.0 and 191.0 μM, respectively. Furthermore, hydroxylated nonaprenylhydroquinone and octaprenylhydroquinone were also found to induce annexin V externalization in K562 cells; it is likely that the main mechanism by which both compounds inhibit cell metabolism and increase the number of apoptotic tumor cells. These compounds exhibited a good activity against K562 cells which will warrant further analysis at the molecular level and offer promising opportunities for the development of new antitumor agents [70].

**Figure 2:**
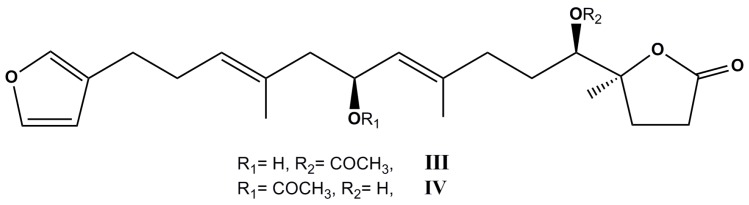
Two new structures 15-acetylirciformonin B (**III**) and 10-acetylirciformonin B (**IV**) isolated from the marine sponge *Ircinia* sp.

One novel terpenoidal natural product, ircinolin A, two new furanoterpene metabolites—15-acetylirciformonin B and 10-acetylirciformonin B (**III** and **IV**, Figure 2), and two known compounds—irciformonin B and irciformonin F, were isolated from an undescribed sponge of the genus *Ircinia*. The cytotoxic activity of the five compounds was determined. 15-Acetylirciformonin B, the most potent of compounds, has exhibited cytotoxicity against the K562, DLD-1, HepG2 and Hep3B cancer cell lines with IC_50_ values of 5.4, 0.03, 0.5, and 1.1 μM, respectively. Furthermore, irciformonin B, 10-acetylirciformonin B, and irciformonin F also were found to exhibit considerable cytotoxicity toward some of the cell lines. It seems that the furan moiety present in these compounds is critical for the cytotoxic activity of the C_22_ furanoterpenoids [71]. The authors have also reported that 10-acetylirciformonin B decreased cell viability through the inhibition of cell growth as well as the induction of DNA damage and apoptosis in a dose-dependent manner. Induction of apoptosis was mediated with the increase in caspases 8, 9 and 3 activation as well as PARP cleavage, indicating that 10-acetylirciformonin B treatment causes apoptosis in leukemia cells, probably, through a caspase dependent regulatory pathway [71].

##### Family: Spongiidae

Sponges belonging to the family Spongiidae include the genera *Spongia*, *Hippospongia*, *Coscinoderma*, *Hyatella*, *Leiosella* and *Rhopaloeides* [72]. As published in the SciFinder database, this group of sponges is known to contain sesquiterpene hydroquinones, diterpenes, furanic and scalarane sesterterpenes, long-chain aliphatic and acetylenic compounds, hepta- and octaprenylhydroquinones, bromotyrosine alkaloids, farnesyl quinols and suvanine analogs, which exhibit cytotoxic, antitumor, antibacterial, antifungal, anti-plasmodial (antimalarial) and protein tyrosine phosphatase 1B (PTP1B) inhibitory activities.

Therefore, eight new sesterterpenes, including structurally related pentaprenylhydroquinones, and seven known ones of the same structural classes, were isolated from an unidentified sponge of the genus *Coscinoderma*. Regarding cytotoxicity against the K562 cell lines, coscinoquinols 1 and 2—with LC_50_ value of 8 µM for both compounds—have shown more potent inhibition than doxorubicin with LC_50_ value of 13.0 µM, while the halisulfates with hydroquinone and furan moieties were inactive. Regarding the suvanine salts, one was again far more active than other with LC_50_ values of 16.0 and 200.0 µM, respectively. The modified furan-bearing derivatives have shown moderate to weak inhibition against K562 cells with LC_50_ values in a range of 15.0 to 59.0 µM [73].

Eight new acyclic manoalide-related sesterterpenes, hippolides A–H, together with two known manoalide derivatives, (6*E*)-neomanoalide and (6*Z*)-neomanoalide, were isolated from *Hippospongia lachne*. Hippolide A has exhibited cytotoxicity against A549, HeLa, and HCT-116 cell lines with IC_50_ values of 5.22 × 10^−2^, 4.80 × 10^−2^, and 9.78 × 10^−2^ µM, respectively. Hippolide B has shown moderate cytotoxicity against the HCT-116 cell lines with IC_50_ value of 35.13 µM [74].

##### Family: Thorectidae

Marine sponges belonging to the family Thorectidae include 23 genera grouped into two subfamilies: Thorectinae—*Aplysinopsis*, *Cacospongia*, *Collospongia*, *Dactylospongia*, *Fascaplysinopsis*, *Fasciospongia*, *Fenestraspongia*, *Hyrtios*, *Luffariella*, *Narrabeena*, *Petrosaspongia*, *Scalarispongia*, *Semitaspongia*, *Smenospongia*, *Taonura*, *Thorecta*, *Thorectandra* and *Thorectaxia*—and Phyllospongiinae—*Candidaspongia*, *Carteriospongia*, *Lendenfeldia*, *Phyllospongia* and *Strepsichordaia* [75]. As demonstrated in the SciFinder database, this family of sponges enclose representatives that biosynthesize sterols, brominated indole alkaloids, sesquiterpene hydroquinones, adenine-related compounds, manoalide-related sesterterpenes and nitrogenous macrolides, with characteristic biological properties including cytotoxic, antibacterial, anti-inflammatory and antidepressant, among other activities.

Thus, the extracts of an undescribed specimen of marine sponge belonging to the genus *Candidaspongia* have shown selective cytotoxicity toward melanoma cells in the NCI-60 anticancer drug cell line screen. Continued investigation of the *Candidaspongia* sp. extracts led to the isolation of three new tedanolide analogs, precandidaspongiolides A and B, and candidaspongiolide B, as well as candidaspongiolide A and tedanolide. Candidaspongiolides A and B were the most potent constituents and have shown low nanomolar activity against several melanoma cell lines [76].

In a previous study, smenospongine, a sesquiterpene aminoquinone isolated from *Dactylospongia elegans* had shown antiproliferative or cytotoxic activities on leukemia cells. In this study, it was found that smenospongine has inhibited proliferation, migration and tube formation of human umbilical vein endothelial cells (HUVEC). Moreover, smenospongine inhibited the growth of 39 human solid cancer cells in vitro, with a mean Log GI_50_ value of −5.55. Therefore, smenospongine exhibits antitumor activity on solid tumors via two mechanisms, an antiangiogenic effect on endothelial cells and direct inhibition of growth of tumor cells [77].

A new sesquiterpene benzoxazole, nakijinol B, its acetylated derivative, nakijinol B diacetate, and two new sesquiterpene quinones, smenospongines B and C, were isolated from the methanol extract of the marine sponge *D. elegans*. The isolated compounds were assessed for their cytotoxicity against a panel of human tumor cell lines (SF-268, H460, MCF-7 and HT-29) and a normal mammalian cell line (CHO-K1). All compounds were found to have activities with GI_50_ values in a range of 1.8 to 46.0 µM and lacked selectivity for tumor versus normal cell lines [78]. Synthesis of benzoxazole was reported by Thomas et al. [39].

Seven new nitrogenous macrolides, designated salarins D–J, closely related to salarins A–C, were isolated from an unidentified marine sponge of the genus *Fascaplysinopsis*. All compounds were evaluated for their cytotoxicity against K562 and UT-7 human leukemia cells. While salarins D, E, H, and J displayed dose- and time-dependent inhibition of proliferation, salarins F and I were not active in these assays [79].

Chemical investigation of *Fasciospongia* sp. returned the new meroterpene sulfate fascioquinol A, together with the known geranylgeranyl 1,4-hydroquinone, which was identified as the dominant cytotoxic principle in the extract of this sponge, with selective inhibitory activity against gastric adenocarcinoma (AGS) cell lines with IC_50_ value of 8.0 µM, and neuroblastoma (SH-SY5Y) cell lines with IC_50_ value of 4.0 µM [80].

**Figure 3:**
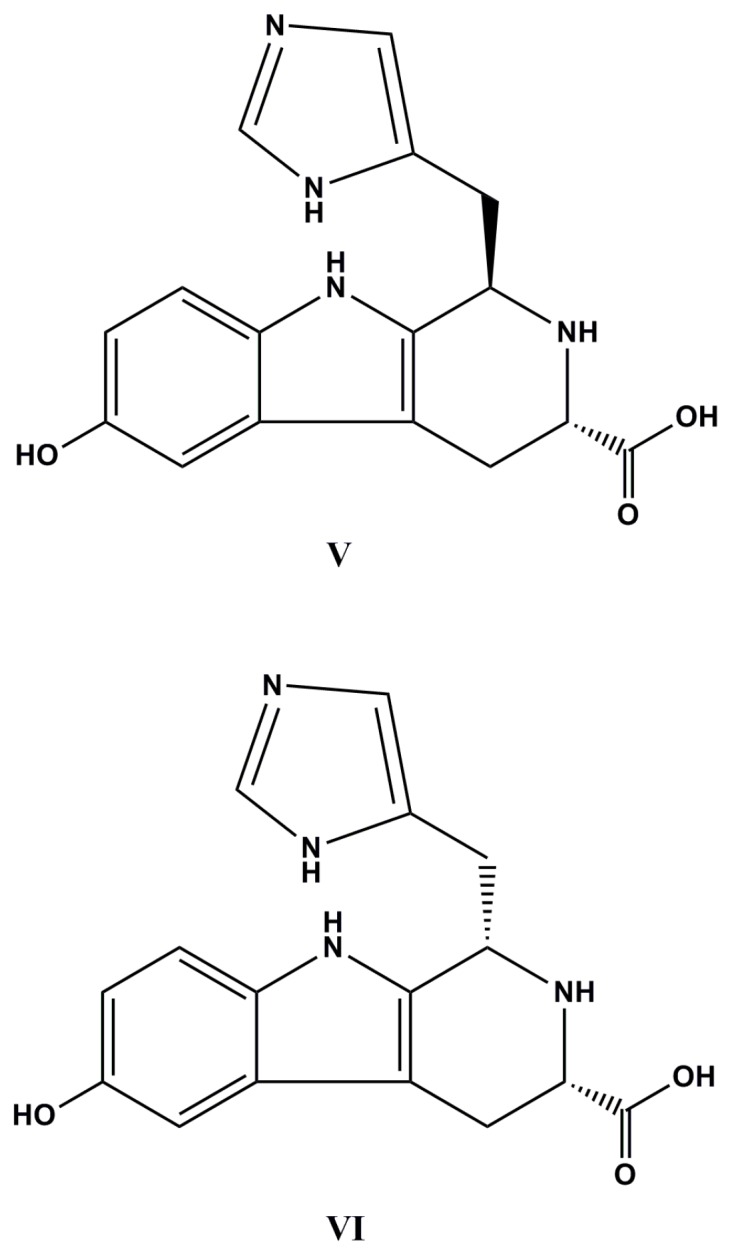
Structures of hyrtioreticulins A (**V**) and B (**VI**) isolated from the marine sponge *Hyrtios reticulatus*.

A new 1-imidazoyl-3-carboxy-6-hydroxy-β-carboline alkaloid, named hyrtiocarboline, was isolated from a Papua New Guinea marine sponge, *Hyrtios reticulatus*. This compound has shown selective antiproliferative activity against H522-T1 non-small cell lung, MDA-MB-435 melanoma, and U937 lymphoma cancer cell lines [81]. In addition, hyrtioreticulins A–E were isolated from other specimen of *H. reticulatus*, along with a known alkaloid, hyrtioerectine B. Hyrtioreticulins A and B (**V** and **VI**, Figure 3) have inhibited ubiquitin-activating enzyme (E1) with IC_50_ values of 0.75 and 11.0 µg·mL^−1^, respectively, measured by their inhibitory abilities against the formation of an E1-ubiquitin intermediate. So far, only five E1 inhibitors, panapophenanthrine, himeic acid A, largazole and hyrtioreticulins A and B, have been isolated from natural sources and, among them, hyrtioreticulins A is the most potent E1 inhibitor [82].

(+)-Spongistatin 1, a macrocyclic lactone isolated in the 90’s from the marine sponge *Hyrtios erecta*, is an extremely potent growth inhibitory agent having activity against a wide variety of cancer cell lines, while exhibiting low cytotoxicity against quiescent human fibroblasts. (+)-Spongistatin 1 was applied in an orthotopic in vivo model of human pancreatic cancer. This compound significantly reduced tumor growth, which correlates with a strong apoptosis induction (DNA-fragmentation) and long-term effects on clonogenic survival of pancreatic tumor cells (L3.6pl) in vitro. In addition, the formation of metastasis was reduced in (+)-spongistatin 1 treated mice [83]. Consistent with a microtubule-targeting mechanism of action, (+)-spongistatin 1 causes mitotic arrest in DU145 human prostate cancer cells, and exhibits significant in vivo antitumor activity in the LOX-IMVI human melanoma xenograft model. According the authors, (+)-spongistatin 1 is, thus, an important class of microtubule targeting anticancer agent [84]. Stereocontrolled total synthesis of (+)-spongistatin 1 has been reported by Smith et al. [85].

**Figure 4:**
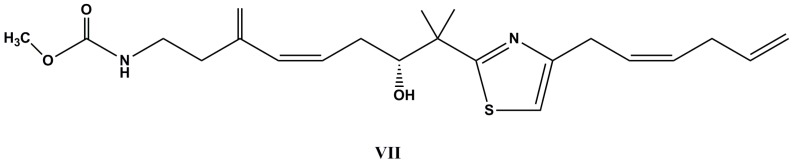
Structure of mycothiazole (**VII**) isolated from *Petrosaspongia mycofijiensis*.

The extract of the marine sponge *Petrosaspongia mycofijiensis* yielded mycothiazole (**VII**, Figure 4), a solid tumor selective compound with no known mechanism for its cell line-dependent cytotoxic activity. Mycothiazole has inhibited hypoxic HIF-1 signaling in tumor cells with IC_50_ value of 1.0 nM, which was correlated with the in vitro suppression of hypoxia-stimulated tumor angiogenesis [86]. A total synthesis of (±)-mycothiazole had been achieved, starting from 2,4-dibromothiazole [87].

6^′^-Iodoaureol, an iodo-sesquiterpene hydroquinone, and 6^′^-aureoxyaureol, a bissesquiterpene hydroquinone, as well as four brominated indole alkaloids were isolated from an unidentified marine sponge of the genus *Smenospongia* (CRI 546) collected from Phi Phi Island, Krabi province, Thailand. Additionally, were also isolated four known sesquiterpene hydroquinones (aureol, 6^′^-chloroaureol, aureol acetate and ent-chromazonarol), ten known brominated indole alkaloids, ergosterol and furospinosulin-1. The compound 5,6-dibromotryptamine has shown good cytotoxicity in MOLT-3 and HeLa cells with IC_50_ values of 5.4 and 9.4 µM, respectively, and has exhibited only moderate activity in HepG2 and HuCCA-1 cells with IC_50_ values of 23.1 and 23.6 µM, respectively. While the 5,6-dibromo-1*H*-indole-3-carboxylic acid methyl ester was moderately cytotoxic in HeLa cells with the IC_50_ value of 13.0 µM. Aureol has shown moderate cytotoxicity in LH-60 cells with IC_50_ value of 14.6 µM, and weak activity in A549 cells with IC_50_ value of 76.4 µM [88]. A concise synthesis of 5,6-dibromotryptamine has been accomplished, starting from the intermediate 5,6-dibromoindole-3-carbaldehyde [89]. First synthesis of the chiral paureol had been described before, starting from (+)-arenarol [90], while other syntheses have been recently reported [91,92].

#### 2.1.5. Order: Haplosclerida

Until recently, in *SP*, Haplosclerida order included the suborders Haplosclerina, Petrosina (both marine) and Spongillina *incertae sedis* (freshwater sponges), which were abandoned. However, because of the lack of combined morphological/molecular phylogenetic evidences in Haplosclerida *sensu stricto*, the genera content of their families remains nowadays unchanged. Therefore, currently this order encompasses three suborders: Haplosclerina with the families Callyspongiidae, Chalinidae and Niphatidae; Petrosina with families Calcifibrospongiidae, Petrosiidae and Phloeodictyidae; and Spongillina with families Spongillidae, Malawispongiidae, Metaniidae, Metschnikowiidae, Palaeospongillidae, Potamolepiidae and Lubomirskiidae. Haplosclerina and Petrosina appear closely related morphologically and are controversial higher taxa [45,93], where natural products have been deteted and isolated only to sponges belonging to three families, namely:

##### Family: Callyspongiidae

The family Callyspongiidae encompasses species belonging to the genera *Arenosclera*, *Callyspongia*, *Dactylia* and *Siphonocalina* [94], which contain sterols, triterpenes, polyketides, polyacetylenes, diketopiperazines and tetracyclic alkylpiperidine alkaloids (compiled from the SciFinder database. These metabolites display a range of biological activities such as cytotoxic, antimicrobial, antifungal and anti-chlamydia, as well as mutagenic and antimutagenic activities, between other effects.

Thus, a bioassay-guided fractionation of the ethyl acetate extract from a unidentified specimen of sponge from the genus *Callyspongia* led the isolation of three polyacetylene metabolites, a new polyacetylene diol named callyspongidiol along with two known compounds, siphonodiol and 14,15-dihydrosiphonodiol. Polyacetylenes have exhibited antiproliferative activity against HL-60 cancer cell lines with IC_50_ values of 6.5, 2.8, and 6.5 µg·mL^−1^, respectively. These metabolites have also induced apoptosis in HL-60 cells [95]. A short synthesis of siphonodiol had been described by Gung et al. [96].

On the other hand, the fractionation of the ethyl acetate extract of the marine sponge *Callyspongia aerizusa* yielded seven new cytotoxic cyclic peptides named callyaerins A–F and H. Callyaerins A, B, D, and F together with callyaerins C and E have shown biological activity in various cytotoxicity assays employing different tumor cell-lines as L5178Y, HeLa, and PC12. Callyaerins E and H have exhibited strong activity against the L5178Y cell lines with ED_50_ values of 0.39 and 0.48 µM, respectively [97].

##### Family: Niphatidae

Members of the family Niphatidae include different species belonging to the genera *Amphimedon*, *Cribrochalina*, *Dasychalina*, *Gelliodes*, *Haliclonissa*, *Hemigellius*, *Microxina*, *Niphates* and *Pachychalina* [98]. The SciFinder database outlines that the metabolic profiling of this family includes sterols, fused aromatics, hexaketides, various types of alkaloids (comprising diamines, polymeric pyridinium compounds, precursors of manzamine, bis-pyridines, beta-carboline compounds, bicyclic amidines and 3-alkylpiperidines), new purines, macrocyclic lactones/lactams and glycerol lipids, which have cytotoxic, anticancer, antibacterial (antituberculosis), antifungal, antimalarial, nematocidal, ichthyotoxic and insecticidal activities.

Thus, two new 3-alkylpyridine alkaloids, pyrinodemins E and F, were isolated from an undescribed sponge of the genus *Amphimedon*. Pyrinodemins E and F are novel 3-alkylpyridine alkaloids possessing a 4-(methoxyamino) piperidinone moiety and an indol-3-glyoxylamide moiety, respectively. Pyrinodemin E has shown in vitro cytotoxicity against P388 and L1210 murine leukemia cells with IC_50_ values of 5.7 and 8.8 µg·mL^−1^, respectively, while pyrinodemin F did not show such activity [99].

Extracts obtained from *Niphates digitalis* have shown strong activity in a cell-based assay designed to detect antagonists of the androgen receptor (AR) that could act as lead compounds for the development of a new class of drugs to treat castration recurrent prostate cancer (CRPC). Assay-guided fractionation has shown that niphatenones A and B, two new glycerol ether lipids, were the active components of the extracts [100]. Pure niphatenone B has shown in vitro cytoxicity against LNCaP prostate cancer cells with IC_50_ value of 7.0 µM, while niphatenone A has shown weaker activity that was not statistically different from that of the negative control. The syntheses of niphatenones A and B have been reported by these same authors.

##### Family: Petrosiidae

Marine sponges belonging to the family Petrosiidae include four valid genera: *Acanthostrongylophora*, *Neopetrosia*, *Petrosia* and *Xestospongia* [101]. As per the SciFinder database, the family Petrosidae is known to contain hydroperoxyl sterols, merosesquiterpenes, sesquiterpenic benzoquinones, polyacetylenic compounds, diverse alkaloids (dopamine, tetracyclic bis-piperidines, carbolines, manzamines and pyrimidin-type alkaloids), galactosyl diacylglycerols, sphingolipids and biscembranoids. These compounds encompass a range of activities such as cytotoxic, anticancer, anti-infective, leishmanicidal, as well as inhibitory activities toward Forkhead box O3a (Foxo3a), 3-hydroxy-3-methylglutaryl CoA reductase gene fluorescent protein (HMGCR-GFP), and nuclear factor kappa B (NF-κB) luciferase, among other bioactivities.

**Figure 5:**
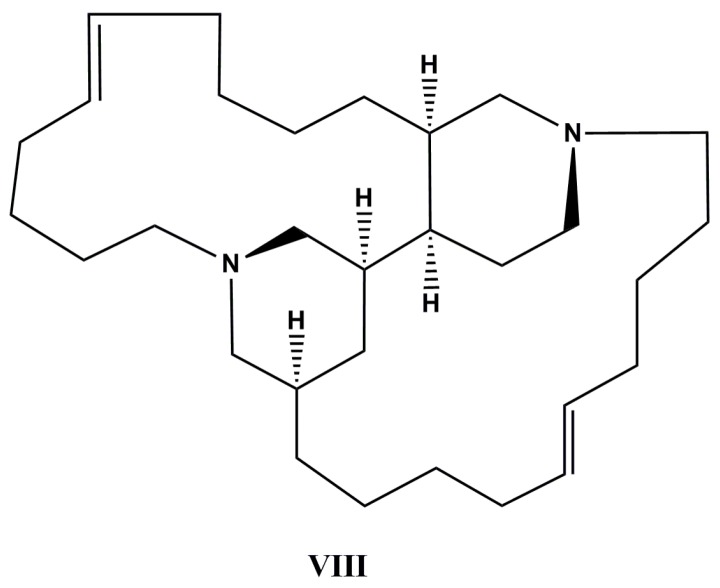
Structure of neopetrosiamine A (**VIII**) isolated from *Neopetrosia proxima*.

A new tetracyclic bis-piperidine alkaloid, neopetrosiamine A (**VIII**, Figure 5), was extracted from *Neopetrosia proxima* collected in the Caribbean Sea. Upon screening in the NCI’s in vitro antitumor assay consisting of 60 human tumor cell lines, neopetrosiamine A has exhibited strong inhibitory activity against MALME-3M melanoma cancer, CCRF-CEM leukemia and MCF7 breast cancer with IC_50_ values of 1.5, 2.0, and 3.5 µM, respectively [102].

Moreover, new miyakosynes A–F were isolated from an unidentified marine sponge belonging to the genus *Petrosia*. Miyakosynes A–D and a mixture of miyakosynes E and F have exhibited cytotoxic activity against HeLa cells with IC_50_ values of 0.10, 0.13, 0.04, 0.15, and 0.30 µg·mL^−1^, respectively [103]. On the other hand, six linear acetylenes, (−)-duryne and (−)-durynes B–F, were isolated from other undescribed specimen of the genus *Petrosia*. (−)-Duryne was found to be the enantiomer of (+)-duryne, a previously reported sponge metabolite. Durynes B–F have shown strong cytotoxicity against HeLa cells with IC_50_ values between 0.08 and 0.50 μM [104]. An efficient enantioselective synthesis of (+) and (−)-duryne had been reported by Gung and Omollo [105].

Manzamine A is a β-carboline alkaloid isolated from *Xestospongia ashmorica* that was suspected to have inhibitory activity against the mitogen-activated protein kinase (MAPK or MAP kinase). As a result, the effects of Manzamine A were studied in pancreatic cancer cells. Manzamine A decreased single cell formation, abrogated cell migration and restored the susceptibility of the cells to TRAIL-induced apoptosis in AsPC-1 cells [38]. Total synthesis of manzamine A was described by Jakubec et al. [106].

#### 2.1.6. Order: Homosclerophorida

The order homosclerophorida formerly was a monotypic order, having the family Plakinidae as its unique representative [107].

##### Family: Plakinidae

The Plakinidae family consists of seven valid genera including *Corticium*, *Oscarella*, *Placinolopha*, *Plakina*, *Plakinastrella*, *Plakortis* and *Pseudocorticium* [107]. As published in the SciFinder database, this group of sponges biosynthesizes diverse structural classes of compounds such as glycosylated sesterterpenes, bacteriohopanoids, steroidal alkaloids (stigmatellins and related compounds), cyclic peroxides with a plakortolide skeleton and non-peroxide plakortin metabolites, polyketide derivatives (polyketide endoperoxides and compounds belonging to spiculoic and to the zyggomphic acid class), thiazine-4-alkylpyridinium and bis-oxygenated pyrroloacridine alkaloids, polypeptides, lysophospholipids, bromopyrroles and bromoaromatic filiformins, amphiasterins, glycolipids, and epidioxymanadic acids, which possess diverse biological activities such as citotoxicity (anticancer), antiangiogenic, anti-HIV, antimicrobial (antifungal and antimycobacterial), DNA- and RNA-cleaving properties, antiprotozoan (antimalarial), schistosomicidal and anti-inflammatory activities. Some of these metabolites act as selective inhibitors of proliferation of HUVECs and activating sarcoplasmic reticulum Ca^2+^–ATP enzymes, showing besides immunosuppressive effects.

Thus, a new cyclic peroxide plakortisinic acid, and a new ketone derivative, in addition to six known compounds—an *α*,β-unsaturated ester, plakortide N, plakortide F and its free acid, plakortone D, and a furan-containing molecule, were isolated from an undescribed Jamaican marine sponge of the genus *Plakortis*. In general plakortide N and plakortide F acid were moderately cytotoxic against the NCI-60 tumor cell lines. In addition, they were remarkably active against colon cancer (KM12 cell lines) and melanoma (LOX-IMVI cell lines), with GI_50_ values in a range of 0.02 to 0.3 μM. Plakortisinic acid was completely inactive against all tumor cell lines [108]. A total synthesis of plakortide E has been reported by Sun et al. [109].

Two novel polyketides, simplextones A and B, were isolated from the marine sponge *Plakortis simplex*. Simplextones A and B have exhibited moderate cytotoxicity against HCT-116 (colon cancer), SGC7901 (gastric cancer), HeLa (cervical cancer), and SW480 (colon cancer) human cell lines using the MTT assay. Simplextone A has exhibited IC_50_ values of 26.3, 57.4, 64.7, and 60.6 µM, respectively, while simplextone B has shown IC_50_ values of 23.7, 45.8, 66.2, and 61.1 µM, respectively [110].

#### 2.1.7. Order: Poecilosclerida

The revised Poecilosclerida remains the largest order, in term of species, and includes 25 families distributed in four sub-orders, namely: sub-order Latrunculina (family Latrunculiidae), sub-order Microcionina (families Acarnidae, Microcionidae, Raspailiidae and Rhabderemiidae), sub-order Mycalina (families Cladorhizidae, Desmacellidae, Esperiopsidae, Guitarridae, Hamacanthidae, Isodictyidae, Merliidae, Mycalidae and Podospongiidae), and sub-order Myxillina (families Chondropsidae, Coelosphaeridae, Crambeidae, Crellidae, Dendoricellidae, Desmacididae, Hymedesmiidae, Iotrochotidae, Myxillidae, Phellodermidae and Tedaniidae) [47]. Despite the large number of species of marine sponges belonging to the order Poecilosclerida, the triennial database search has detected only new natural citotoxics for representatives of the families Acarnidae, Coelosphaeridae, Hymedesmiidae, Latrunculiidae and Podospongiidae, which can be seen hereafter.

##### Family: Acarnidae

Sponges belonging to the family Acarnidae include species of the genera *Acanthorhabdus*, *Acarnus*, *Acheliderma*, *Cornulella*, *Cornulum*, *Damiria*, *Dolichacantha*, *Iophon*, *Megaciella*, *Paracornulum*, *Tedaniphorbas*, *Wigginsia* and *Zyzzya* [47]. Despite the high number of species of this family, some genera are up till now poorly explored. However, between the studied species, were isolated diverse ketosteroids, alkaloids (bispyrroloiminoquinones and pyrroloquinoline alkaloids of the makaluvamine family), fatty acids and cyclic peroxides and guanidines, which display a range of pharmacological properties such as cytotoxic (anticancer), antiviral, antimicrobial (antibacterial), antimalarial and as well as antioxidant activities (compiled from the SciFinder database).

**Figure 6:**
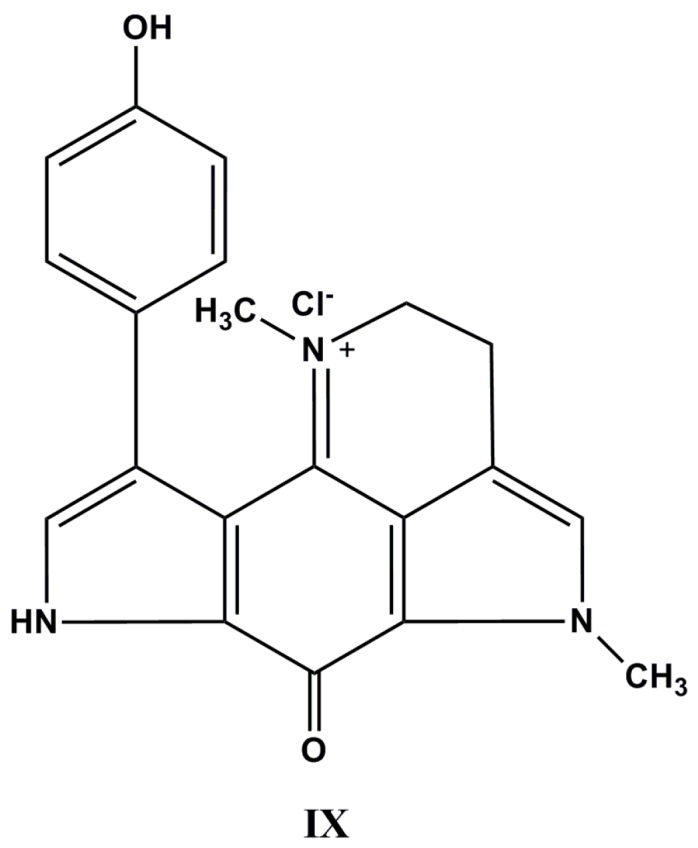
Tsitsikammamine C (**IX**) isolated from a marine sponge *Zyzzya* sp.

A new bispyrroloiminoquinone alkaloid named tsitsikammamine C (**IX**, Figure 6) has displayed selectivity indexes of >200-fold against HEK293 cells. Earlier reported compounds makaluvamines G, J, K, L and damirones A and B were also isolated from an undescribed sponge of the genus *Zyzzya*. Makaluvamines G, J, and L have displayed moderate cytotoxicity against HEK293 cells with IC_50_ values in the range of 1.0 to 4.0 µM [111]. The total synthesis of makaluvamines was previously achieved [112].

##### Family: Coelosphaeridae

Sponges belonging to the family Coelosphaeridae include the genera *Celtodoryx*, *Chaetodoryx*, *Coelosphaera*, *Forcepia*, *Histodermella*, *Inflatella*, *Lepidosphaera* and *Lissodendoryx* [47]. The SciFinder database lists this group of sponges as known to contain sterols, sulfated exopolysaccharides with antiviral activity, lectins, polyketide-derived macrolides with cytotoxic activity, a pigment biogenetically derivable from tryptamine and tyramine which also displays cytotoxicity to several tumor cell lines, and it is a moderate inhibitor of topoisomerase-I, DNA, RNA and protein synthesis, and finally, the well-known anticancer molecule halichondrin B.

In accordance with the above-mentioned methods, two new dimeric sterols named manadosterols A and B were isolated from *Lissodendryx fibrosa*. Both sterols have inhibited the Ubc13-Uev1A interaction at IC_50_ values of 0.09 and 0.13 µM, respectively, and were more potent than leucetamol A—the first such inhibitor isolated from another marine sponge [113].

##### Family: Crambeidae

Marine sponges of the family Crambeidae include species of the genera *Crambe*, *Discorhabdella*, *Lithochela* and *Monanchora* [47]. The SciFinder database shows this family of sponges as encompassing sesterterpenoids (phorbaketals), bicycle [4.3.1] steroids, and acyclic and pentacyclic guanidine alkaloids, which possess cytotoxic, antibacterial, antifungal, antiviral, and antiprotozoal (antiplasmodium) activities. Some of these metabolites show besides interesting anti-inflammatory properties.

Thus, five guanidine alkaloids—mirabilin B, 8β-hydroxyptilocaulin, ptilocaulin, and a mixture of the 8β- and 8*α*-epimers of 8-hydroxymirabilin—were isolated from *Monanchora arbuscula* colonies collected off the Northeastern Brazilian coast. The cytotoxicity of the isolated compounds was evaluated against four tumor HL-60, HCT-8, MDA-MB-435, and SF-295 cell lines—showing that both epimers were inactive, while 8β-hydroxyptilocaulin and ptilocaulin presented IC_50_ values in the range of 7.9 to 61.5 µM, and 5.8 to 40.0 µM, respectively. Further studies on the mechanism of action of ptilocaulin—using HL-60 leukemia cells—have demonstrated that this guanidine compound induces apoptosis of the treated cells [114]. The stereospecific total synthesis of (±)-ptilocaulin has been achieved [115].

On the other hand, monanchocidin, a new guanidine alkaloid with an unprecedented skeleton system was isolated from *Monanchora pulchra*. Monanchocidin has shown cytotoxic activities on cancer cell lines of various tissues including human leukemia THP-1 with IC_50_ value of 5.1 μM, human cervix epithelioid carcinoma HeLa with IC_50_ value of 11.8 μM, and mouse epidermal JB6 Cl41 cell lines with IC_50_ value of 12.3 μM. It also induces 66% of early apoptosis in THP-1 cells at 3.0 μM concentration [116].

**Figure 7:**
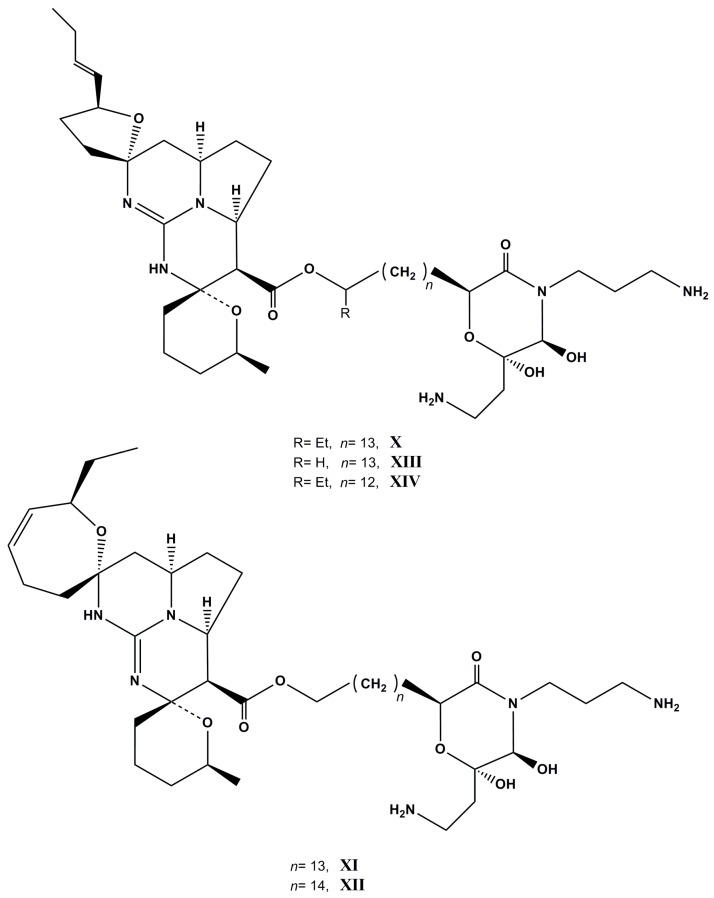
Monanchocidin A (**X**) and monanchocidins B–E (**XI**–**XIV**) isolated from *Monanchora pulchra*.

Also, new unusual polycyclic guanidine alkaloids monanchocidins B–E (**XI**-**XIV**, Figure 7), along with the known monanchocidin A (**X**), were isolated from another exemplar of *M. pulchra*. All five compounds have shown potent cytotoxic activities against HL-60 human leukemia cells with IC_50_ values of 0.54, 0.20, 0.11, 0.83, and 0.65 µM, respectively [117]. Monanchocidin A contains an unusual highly oxidized morpholinone fragment. The synthesis of this heterocyclic scaffold has been recently confirmed [118]. Monanchomycalins A and B have exhibited potent cytotoxic activities against HL-60 human leukemia cells with IC_50_ values of 0.12 and 0.14 µM, respectively [119]. Enantioselective synthesis of the monanchocidin A is in progress [120].

##### Family: Hymedesmiidae

Marine sponges belonging to the family Hymedesmiidae are possibly of polyphyletic nature and include the genera *Acanthancora*, *Hamigera*, *Hemimycale*, *Hymedesmia*, *Kirkpatrickia*, *Myxodoryx*, *Phorbas*, *Plocamionida*, *Pseudohalichondria* and *Spanioplon* [47]. As per the SciFinder database, this family of sponges contain terpenes (hamigerans diterpenoids, gagunin diterpenoids, polyoxygenated diterpenes, tetracyclic diterpenes, diterpenoid pseudodimers and phorbaketals-type sesterterpenoids), steroids (sulfated dimeric and N-imidazolyl steroids), alkaloids (pyridopyrrolopyrimidines, chlorinated phenylpyrrolyloxazoles, benzylidene-2-aminoimidazolones and indole-carboxaldehydes), phenylmethylene hydantoins, brominated benzocyclooctanes, macrolide glycosides, halogenated phenols and aliphatic ketones. These natural products exhibit a range of activities such as cytotoxic (antitumor), anti-invasive and antimetastatic activities. They also show antiviral (against herpes and polio viruses) and antifungal properties, some of them functioning as cyclin-dependent kinase and isocitrate lyase inhibitors, as well as active pharmacological agents against osteoporosis and obesity.

**Figure 8:**
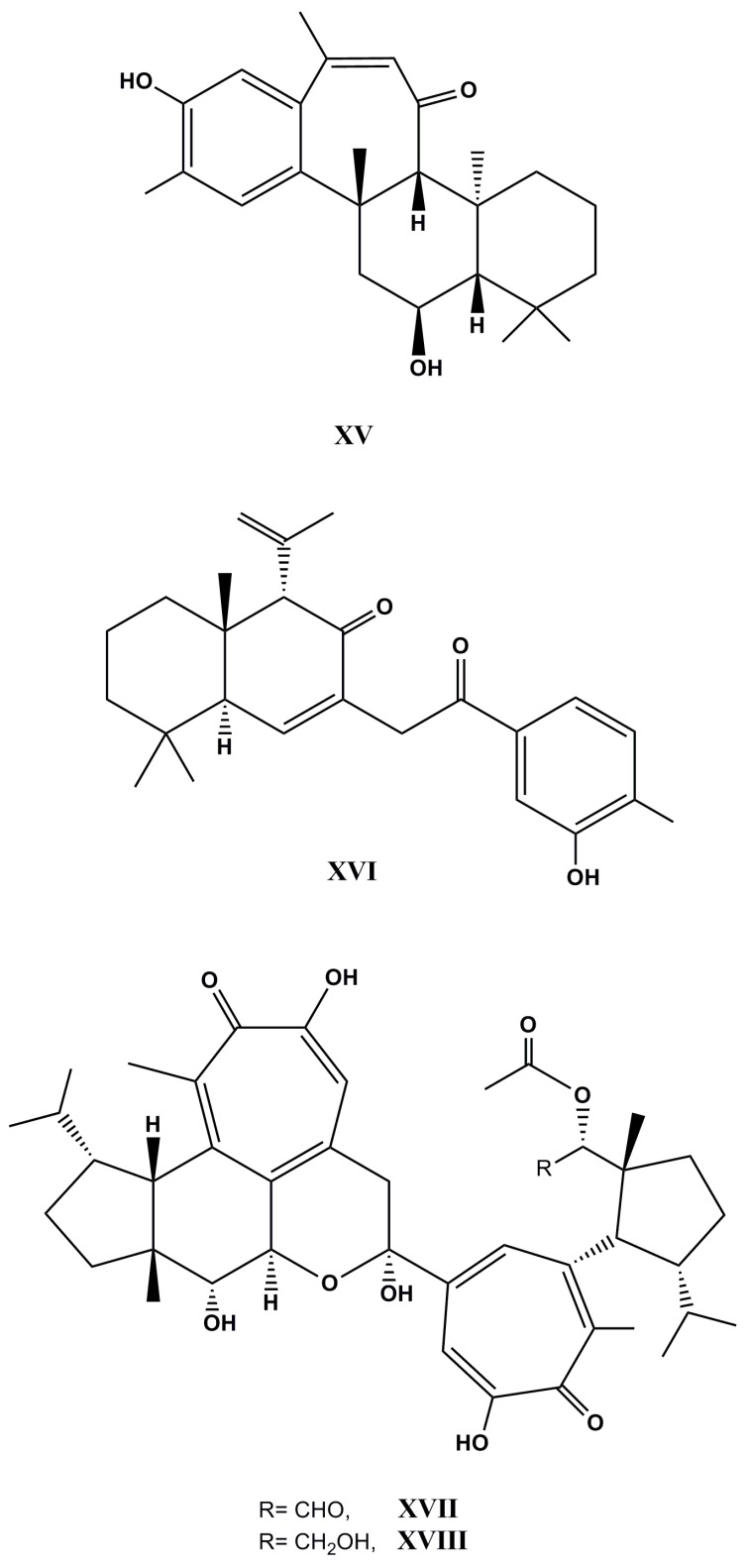
Phorone A (**XV**), isophorbasone A (**XVI**), and gukulenins A and B (**XVII**–**XVIII**).

The chemical investigation of an unidentified marine sponge of the genus *Phorbas* yielded unprecedented sesterterpenoids—phorone A and isophorbasone A (**XV** and **XVI**, Figure 8)—along with ansellone B and phorbasone A acetate. Both compounds have exhibited potent inhibitory activity on nitric oxide production in RAW264.7 lipopolysaccharide-activated mouse macrophage cells with IC_50_ values of 4.5 and 2.8 µM, respectively [121].

On the other hand, gukulenins A and B (**XVII** and **XVIII**, Figure 8) were isolated from the Korean marine sponge *Phorbas gukulensis*. Gukulenins A and B have exhibited potent activities against human pharynx cancer FaDu (IC_50_ value of 57.0 nM and 0.63 μM), colon cancer HCT-116 (IC_50_ value of 62.0 nM and 0.55 μM), renal cancer SN12C (IC_50_ value of 92.0 nM and 0.61 μM), and stomach cancer MKN45 (IC_50_ value of 0.13 μM and 0.72 μM) cell lines [122].

##### Family: Latrunculiidae

Chemical investigation on marine sponges belonging to the family Latrunculiidae, which include species of the genera *Cyclacanthia*, *Latrunculia*, *Sceptrella*, *Strongylodesma* and *Tsitsikamma* [47] afforded decalactones, new terpenes (norsesterterpene peroxides), alkaloids (makaluvic acids, dihydrodiscorhabdin B, discorhabdin, and pyrroloiminoquinone alkaloids of the discorhabdin class), 2-thiazolidinone-containing macrolides and callipeltin-related acyclic peptides (compiled from SciFinder database). This group of sponges presents selective antimicrobial, antifungal, antiprotozoal (antimalarial) activities along with cytotoxic, antitumor and anti-HCV properties. Moreover, they displayed topoisomerase inhibition, cancer chemopreventive potential and inhibitory activity against sortase A, among other properties.

Then, it was isolated from a *Sceptrella* species, two new pyrroloiminoquinone alkaloids of the discorhabdin class, (−)-3-dihydrodiscorhabdin D and (−)-discorhabdin Z, along with 12 known compounds including one previously described synthetic derivatives of the same and related skeletal classes. These compounds have exhibited moderate to strong cytotoxicity against the K562 erythroleukemia cell line, comparable to doxorubicin [123].

##### Family: Podospongiidae

Sponges belonging to the family Podospongiidae include species of the genera *Diacarnus*, *Diplopodospongia*, *Negombata*, *Neopodospongia*, *Podospongia*, *Sceptrintus* and *Sigmosceptrella* [47]. According with the current search database for bioactives (SciFinder), the aforementionated genera of sponges produce a wide range of terpenoids (chlorinated polyfunctional diterpenoids, norditerpene peroxides, norsesterterpene peroxides), latrunculeic acid and latrunculol derivatives, macrolides containing a thiazolidinone moiety, ceramides, diglyceride esters, sphingolipids, poliketides and pyrroloiminoquinones. These compounds have shown cytotoxic, antitumor (anticancer), antineoplastic, anti-inflammatory, antiviral, antimicrobial, antimalarial, antitrypanosomal and antiepileptic activities.

Six norterpenes including negombatoperoxides A and B, the inseparable epimers negombatoperoxides C and D, negombatodiol, and negombatolactone, in combination with three known compounds, (+)-nuapapuin B, (+)-nuapapuin B methyl ester, and (+)-aikupikoxide C, were isolated from *Negombata corticata*. The data have revealed that (+)-nuapapuin B was cytotoxic toward MDAMB-231, MCF-7, HepG2, Hep3B, and A-549 cancer cell lines with IC_50_ values of 0.3, 5.9, 0.9, 41.3 and 0.6 μM, respectively, while its methyl ester has shown activity against the above cancer cell lines with IC_50_ values of 3.5, 38.5, 2.9, 23.7 and 5.38 μM, respectively. However, (+)-aikupikoxide C has shown weaker cytotoxicity toward three of the above cancer cell lines with IC_50_ values ranging from 11.9 to 36.7 μM. The others tested compounds were not cytotoxic (IC_50_ > 50.0 μM) or inactive (negombatodiol and negombatolactone) toward the mentioned five cancer cell lines [124].

#### 2.1.8. Order: Scopalinida

The order Scopalinida is a monotypic order, having the family Scopalinidae as its unique representative [47].

##### Family: Scopalinidae

Sponges of the novel family Scopalinidae include species belonging to the genera *Scopalina*, *Svenzea* and *Stylissa*—a former halichondrid [47]. As listed in the SciFinder database, this group of sponges contain a variety of interesting secondary metabolites such as brominated fatty acids, sterols (type polyhydroxylated and 5(6→7) abeo-sterols), nitrogenous terpenoids, diterpenes of the isoneoamphilectane class, alkaloids, bromopyrroles, peptides (proline-rich cyclic octapeptides and others) and sphingoglycolipids. These bioactive compounds act as cytotoxic, antimicrobial, antibacterial, antifungal, antitumor (anticancer), anti-inflammatory, and antiplasmodial agents, presenting besides antiproliferative and protein kinase inhibitory activities.

**Figure 9:**
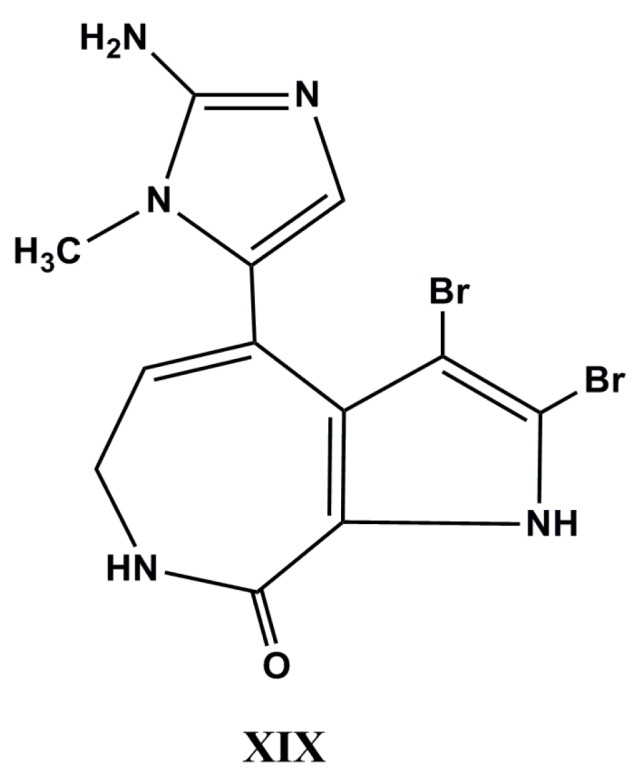
12-*N*-methyl stevensine (XIX) isolated from the marine sponge *Stylissa* sp.

Thus, the chemical investigation of an undescribed marine sponge of the genus *Stylissa* afforded four new brominated alkaloids, including 12-*N*-methyl stevensine (**XIX**, Figure 9), 12-*N*-methyl-2-debromostevensine, 3-debromolatonduine B methyl ester, and 3-debromolatonduine A together with eight known alkaloids identified as (*Z*)-hymenialdisine, (*Z*)-debromohymenialdisine, stevensine, 2-debromostevensine, 3-bromoaldizine, 3,4-dibromopyrrole-2-carbamide, latonduine A, and latonduine B methyl ester, respectively. The results indicated that only 12-*N*-methyl stevensine, (*Z*)-hymenialdisine, (*Z*)-debromohymenialdisine, and latonduine have shown high in vitro cytotoxicity against mouse lymphoma L5187Y cell lines with EC_50_ values of 3.5, 1.8, 2.1 and 9.0 µg·mL^−1^, respectively [125]. The synthesis of hymenialdisine has been described by Saleem and Tepe [126].

#### 2.1.9. Order: Suberitida

The order Suberitida has the families Halichondriidae, Stylocordylidae and Suberitidae as its representatives [47].

##### Family: Halichondriidae

The family Halichondriidae is considered not monophyletic and encompasses 11 genera named *Amorphinopsis*, *Ciocalapata*, *Ciocalypta*, *Cryptax*, *Halichondria*, *Hymeniacidon*, *Johannesia*, *Laminospongia*, *Sarcomella*, *Spongosorites* and *Topsentia*, being the latter genus considered as Suberitida *incertae sedis* [47]. Furthermore, this group of sponges is known to contain a wide range of bioactive metabolites such as steroids (cholesterol sulfate, new sulfated sterols and isopropylated peroxides), polyacetylenic alcohols, isonitrile diterpenes (amphilectanes), macrocyclic polyethers, nitrogenous sesquiterpenes, dimeric sesquiterpenoids, fatty acids (oxylipins and brominated fatty acids), biindole pigments, alkaloids [discorhabdin class, alkylpyridines, tetracyclic bipiperidines, mono- and bisindole compounds (of the topsentin and hamacanthin classes) and imidazolediylbis-indoles], polyketide phosphodiesters, trisoxazole-containing macrolides, and guanidinic compounds among others (SciFinder database).

These compounds are useful as potential therapeutics, particularly, due their antioxidant, cytotoxic (anticancer), anti-inflammatory, antimicrobial (antibacterial and antifungal), antiviral (HIV- and herpes-inhibitory), and antiparasitic (anti-plasmodium and antimalarial) activities. Some of these bioactives functioning as immunostimulants, reduce cholesterol uptake and, also, act in the basolateral secretion and ACAT-2 mRNA expression and increase the expression of ABCA1 mRNA in Caco-2 cells.

Thus, novel sesquiterpene alkaloids, halichonines A–C, were identified from the marine sponge *Halichondria okadai*. All three halichonine congeners have shown moderate growth-inhibitory activities against mammalian cancer cells lines (L1210 and PC13) using the MTT assay. Halichonine B was then subjected to the trypan blue dye exclusion using HL-60 human leukemia cells, and has shown cytotoxicity at IC_50_ value of 0.6 µg·mL^−1^. DNA ladder analysis revealed that halichonine B has induced DNA fragmentation in HL-60 cells [127].

#### 2.1.10. Order: Tetractinellida

The re-classification based on molecular results of Demospongiae class suggested by Morrow and Cárdenas [47], between other modifications, propose to resurrect or upgrade six orders from *SP* including the Tetractinellida order that involves, among others, 11 former lithistid families namely: Ancorinidae, Azoricidae, Calthropellidae, Corallistidae, Geodiidae, Isoraphiniidae, Macandrewiidae, Neopeltidae, Pachastrellidae, Phymaraphiniidae, Phymatellidae, Pleromidae, Samidae, Scleritodermidae, Siphonidiidae, Spirasigmidae, Tetillidae, Theonellidae, Theneidae, Thoosidae, Thrombidae and Vulcanellidae. Bioactive metabolites isolated from sponges belonging to certain Tetractinellid families are detailed below:

##### Family: Ancorinidae

Marine sponges belonging to the Ancorinidae family include 15 valid genera (of 37 nominal ones) namely *Ancorina*, *Asteropus*, *Chelotropella*, *Cryptosyringa*, *Dercitus*, *Disyringa*, *Ecionemia*, *Holoxea*, *Jaspis*, *Psammastra*, *Stelletta*, *Stellettinopsis*, *Stryphnus*, *Tethyopsis* and *Tribrachium* [47,128].

Despite the high number of species of this family, some genera are up till now little explored. However, of the studied species, were isolated diverse monocyclic diterpene-benzenoids, triterpenes (isomalabaricane-type triterpenoids and triterpenoid oligoglycosides), sterol derivatives, alkaloids (bromotyrosine derivatives, acridine and pentacyclic pyridoacridine compounds, indolizidine and bisguanidinium compounds, and indolo-[3,2-*a*]carbazoles), swinholide polyketides, cyclodepsipeptides, diketopiperazines, monoacylglycerols, glycerol ethers, acetylenic acids, very long-chain methoxylated Δ5,9 fatty acids, lysophosphatidylcholines, sphingolipid hybrid molecules, apocarotenoids, macrolide lactams and amino acid derivatives (SciFinder database). These bioactives have shown interesting pharmacological properties such as cytotoxic (anticancer and antitumoral), antiviral (HIV-Inhibitory), antimicrobial (antibacterial and antifungal), antileishmanial and anthelmintic activities, they also are inhibitors of mitotic spindle formation, inhibitors of protein kinase activity and inhibitors of topoisomerases.

**Figure 10:**
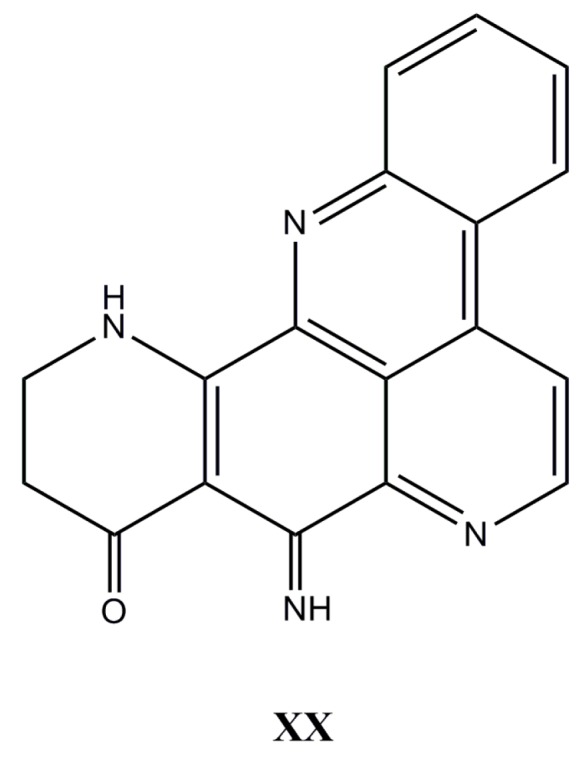
Ecionine A (**XX**) isolated from *Ecionemia geodides*.

Chemical studies of the Australian marine sponge *Ecionemia geodides* resulted in the isolation of two new pyridoacridine alkaloids, named ecionines A and B, along with the previously isolated compounds, biemnadin and meridine. Both novel ecionines contain an imine moiety, which is very rarely found in the pyridoacridine class. All compounds were tested against a panel of human bladder cancer cell lines—the increasingly metastatic TSU-Pr1 series (TSU-Pr1, TSU-Pr1-B1, and TSU-Pr1-B2), and the superficial bladder cancer (5637) cell lines. Ecionine A (**XX**, Figure 10) has displayed potent cytotoxicity against all cell lines, with IC_50_ values ranging from 3.0 to 7.0 µM [129].

From an unidentified marine sponge of the genus *Stelletta*, collected from the west side of Jamieson Reef, Bonaparte Archipelago, North West Western Australia, a new diketopiperazine (DKP) named cyclo-(4-*S*-hydroxy-*R*-proline-*R*-isoleucine) was isolated together with the known bengamides A, F, N, Y, and bengazoles Z, C4, and C6. The novel DKP was proposed to be the product of an enzymatically controlled condensation reaction between D-isoleucine and 4-*S*-hydroxy-d-proline and was not cytotoxic against the cell lines MCF-7, H460, HT-29, SF-268 or CHO-K1 [130].

##### Family: Calthropellidae

Marine sponges of the family Calthropellidae include species of the genera *Calthropella*, *Corticellopsis*, *Pachataxa* and *Pachastrissa* [131]. Although the metabolic profile of this wide group of metazoans is still little understood, some trisoxazole macrolides, anhydrophytosphingosines, lactones, bengamides and bengazole derivatives were isolated (compiled from SciFinder database). Among other properties, these compounds have shown cytotoxic and antiplasmodial activities.

**Figure 11:**
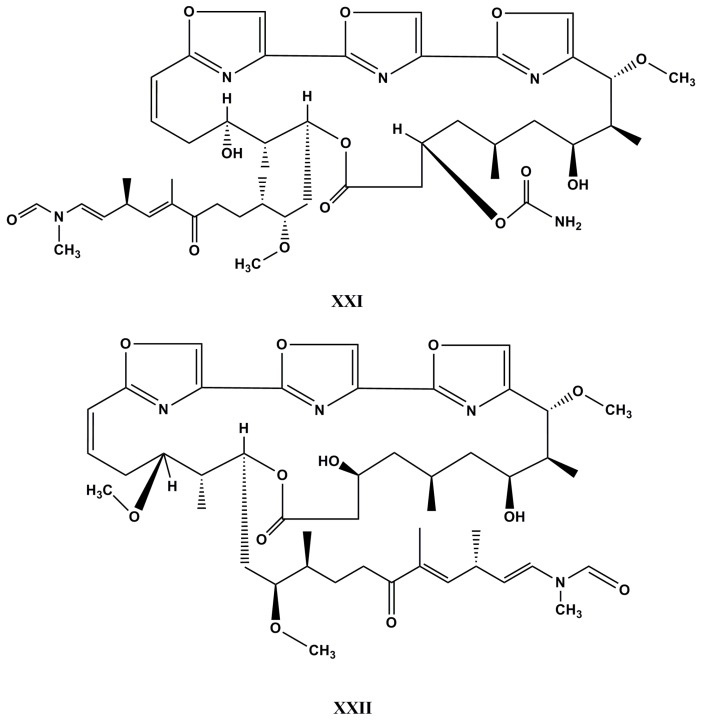
Kabiramides J (**XXI**) and K (**XXII**) isolated from the marine sponge *Pachastrissa nux*.

Thus, in the search for new compounds, three trisoxazole macrolides possessing a 30-α,β-enone moiety, including the known kabiramide G and the new kabiramides J and K (**XXI** and **XXII**, Figure 11) were isolated from the sponge *Pachastrissa nux*, along with the previously reported kabiramides B, C, and D. All the isolated compounds were assayed for cytotoxic activities against MCF-7 breast adenocarcinoma and normal human fibroblast. The compounds have shown strong activities in both models with IC_50_ values in a range of 0.02 to 0.45 µM and 0.05 to 2.37 µM, respectively, except for kabiramide G which has shown only strong cytotoxicity against both cancer cells with IC_50_ values in a range of 0.47 to 7.59 µM, and against normal cell lines at sub- and micromolar concentrations [132].

##### Family: Geodiidae

Sponges belonging to the family Geodiidae include diverse species from the genera *Caminella*, *Caminus*, *Erylus*, *Geodia*, *Melophlus*, *Pachymatisma*, *Penares* and *Rhabdastrella* [133,134] whose systematic has been recently revised based on the reevaluation of their morphological characters and molecular analysis [135]. Although the metabolic profiling of some genera of this group of metazoans is still poorly understood, and their potential biological activities are currently under scrutiny, some classes of biologically active metabolites such as nor-sesquiterpene carboxylic acids, saringosterol, steroidal glycosides, triterpenes (lanostanes, nor-lanostane saponins and isomalabaricanes), longer chain penasterol oligosaccharides, alkaloids (penarolide sulfates), brominated cyclodipeptides and depsipeptides, trisoxazole macrolides, tetramic acid glycosides, glycolipids and unusual amino-acids and fatty acids, were isolated (SciFinder database).

These bioactives have shown a range of biological activities such as cytotoxic and antitumor (anticancer), antimicrobial (antibacterial and antifungal), and antiplasmodial properties; showing in vitro inhibition of human platelet aggregation and producing hemolysis of human erythrocytes. Some aforementioned compounds act as part of the IKK complex in the conventional pathway of NF-kappa-B activation and phosphorylate inhibitors of nuclear factor kappa-B kinase subunit beta (IKK-β) kinase. Also, show 26S proteasome proteolytic activity and the ability to promote stabilization of the DNA-polymerase beta covalent binary complex, functioning as inhibitors of histamine release (*α*-glucosidase inhibitors) (SciFinder database).

**Figure 12:**
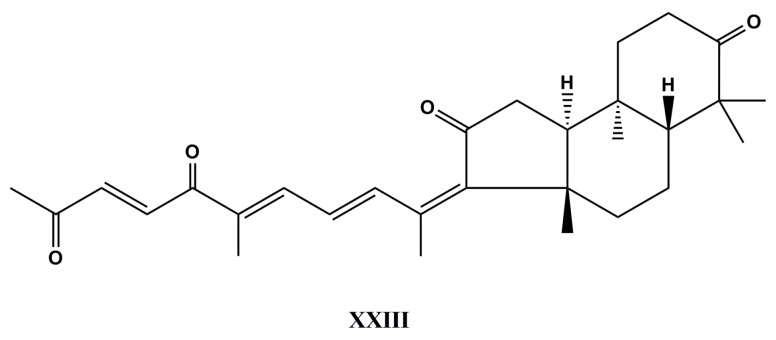
Geoditin A (**XXIII**) isolated from *Geodia japonica*.

Thus, geoditin A (**XXIII**, Figure 12), an isomalabaricane triterpenoid found in many genera of marine sponges has received special pharmacological attention because it inhibited cyclin-dependent kinase activity and subsequently suppressed tumor cell proliferation [136]. This naturally occurring compound was isolated from the marine sponge *Geodia japonica* collected from the South China Sea [137]. According to Cheung et al., after treatment with geoditin A for 24 h, it was observed fragmentation of Golgi structure, suppression of transferrin receptor expression, production of oxidants, and DNA fragmentation in human colon cancer HT29 cells [138], and was also demonstrated to induce apoptosis in leukemia HL-60 cells with IC_50_ value of 3.0 µg·mL^−1^ [136]. Further, it was observed that the cytotoxic effect of geoditin A is likely mediated by a NAC-inhibitable oxidative stress [139].

On the other hand, geoditin A at sublethal doses (≤5.0 µg·mL^−1^) decreased melanogenesis and glycosylation of tyrosinase (TYR) in murine B16F10 melanoma cells (CRL6475, ATCC) in a dose-dependent manner, but ROS- and MITF- in an independent one. Therefore, there is potential for the application and development of this marine compound as a skin-lightening agent [139].

Two new unusual bromine-containing alkaloids were isolated from an unidentified marine sponge belonging to the genus *Penares*, which was collected from Vietnam waters, the South China Sea. Cytotoxic activities of the isolated compounds against tumor cell lines HL-60 and HeLa were determined using MTS reduction into its formazan product. The first compound named 3,11-dibromo-13*H*-indolo[3,2-k]phenanthridine was inactive, while the second compound named 7-bromo-1-(6-bromo-1*H*-indol-3-yl)-9*H*-carbazole has demonstrated moderate inhibition of both cell lines with IC_50_ values of 16.1 and 33.2 µM, respectively [140]. Progress towards the total synthesis of the natural alkaloid 3,11-dibromo-13*H*-indolo[3,2-k]phenanthridine is being still carried out [141].

Thus, from the marine sponge *Rhabdastrella globostellata*, which was collected from Hainan Island in the South China Sea, were isolated nine new isomalabaricane-type triterpenoids namely globostelletins J–R together with jaspolide F, rhabdastrellic acid A, (−)-stellettin E, and stellettins C and D. The authors demonstrated that all the compounds have induced inhibitory activities in human lung adenocarcinoma (A549), human gastric gland carcinoma (BGC-823), human intestinal adenocarcinoma (HCT-8) and human hepatocellular carcinoma (Bel-7402) cells. Besides, HL-60 cells treated for 24 h with 5.0 μM of rhabdastrellic acid A have induced the externalization of phosphatidylserine, which is characteristic of apoptotic cell death [142,143].

On the other hand, seven new isomalabaricane derivatives, rhabdastins A–G and a new monocyclic triterpene glycoside, named rhabdastoside A, were isolated from the methanolic extract of the same species of marine sponge, *R. globostellata*, collected at Amami-Oshima, Japan. Three of them were isolated as their corresponding methyl esters. The isolated compounds possessing a cyclopentane side chain have exhibited weak activity against leukemia HL-60 cells, while compounds with a 2-substituted-propanoate side chain were inactive at growth inhibitory concentration of 100.0 µM [144].

#### 2.1.11. Order: Verongida

The revised classification of Demospongiae class proposed by Morrow and Cárdenas [47] has retained seven orders from 13 ones originally present in the *SP*, including the order Verongida [145]. Therefore, Verongida order involves four families namely Aplysinellidae, Aplysinidae, Ianthellidae, and Pseudoceratinidae.

As outlined in the SciFinder database, Verongid sponges are characterized by a unique biochemistry as the lack terpenes and a great percentage of sterols—generally with the aplystane skeleton—and, especially, elaborate a series of brominated metabolites derived from tyrosine that are considered to be peculiar to species belonging to this order [146]. Some bioactive metabolites from species belonging to three families of this order are detailed below:

##### Family: Aplysinellidae

Members of the family Aplysinellidae encompass three different genera: *Aplysinella*, *Porphyria* and *Suberea* [147], which contain punpehenone-related and bromotyrosine-derived metabolites with varied biological activities as cytotoxic, antiviral, antifungal and inmunomodulatory affects.

From an undescribed marine sponge of the genus *Suberea* were isolated two new brominated compounds, subereaphenol K and 2-(3,5-dibromo-1-ethoxy-4-oxocyclohexa-2,5-dien-1-yl) acetamide, together with subereaphenol B (methyl 2-(2,4-dibromo-3,6-dihydroxyphenyl) acetate) with a revised structure, and five dibromotyrosine-derived metabolites. Some of the compounds as the subereaphenol B and the dibromotyrosine derivatives, aeroplysinin-1 and -2, have exhibited various weak or moderate cytotoxic activities against NIH-3T3, HepG2 and HT-29 cell lines [148]. The synthesis of aeroplysinin-1 had been previously accomplished [149].

##### Family: Ianthellidae

Sponges belonging to the family Ianthellidae include species from the genera *Anomoianthella*, *Hexadella* and *Ianthella* [150], which are known to contain sterols (as petrosterol), sulfated carotenoids, cyclic peptides (bastadins), trimeric hemibastadin congeners, brominated tyrosine derivatives, iantherans, benzofuranic compounds, octopamine derivatives, indole-aldehydes, phenylacetic acids, thymidines, deoxycytidines, araplysillin-I N20-sulfamate and brominated macro-dilactams. These metabolites possess interesting biological activities such as cytotoxic (anticancer), antibacterial and antitrypanosomal, along with inhibitory activity against H^+^/K^+^–ATPase.

Therefore, the focus of the study performed by Calcul et al. was on the bastadin class of bromotyrosine derivatives, isolated from the marine sponge *Ianthella reticulate* as the first report on this category of secondary metabolites. Additionally, two new bastadins were isolated, (*E*,*Z*)-bastadin 19—a diastereoisomer of the known (*E*,*E*)-bastadin 19, and an unusual dibenzo-1,3-dioxepine. The known bastadin 4 has shown strong cytotoxic activity against HCT-116 colon cancer cells with IC_50_ value of 1.28 µM [151]. Total synthesis of dioxepine bastadin 3 has been performed by Pérez-Rodríguez et al. [42] while the synthesis of bastadins in heterogeneous phase, starting from dopamine, has been reported by Decamps et al. [152].

##### Family: Pseudoceratinidae

Marine sponges belonging to the family Pseudoceratinidae include four nominal genera, however, only the genus *Pseudoceratina* is considered valid [153]. This family affords a range of metabolites such as halogenated derivatives (brominated phenols and bromotyramine), alkaloids (spermidine derivatives), cyanoformamide, glycolipid analogs and other compounds. These metabolites have shown cytotoxic, anticancer, antibiotic (antibacterial), antifungal and antimalarial activities.

Thus, a bioassay-directed fractionation of *Pseudoceratina arabica*, using the wound-healing protocol, resulted into the isolation of three known alkaloids, subereamolline A, aerothionin and homoaerothionin. Subereamolline A potently has inhibited the migration and invasion of the highly metastatic human breast cancer cells MDAMB-231 at nanomolar doses [154]. The first total synthesis of (+)- and (−)-subereamollines A and B was reported, and the enantiomeric forms of these natural products were obtained by preparative chiral HPLC separation of the corresponding racemates [41].

### 2.2. Miscellaneous

In support of this review, some remaining families/species of marine sponges were summarized in Table 1, as well as their cytotoxic properties.

## 3. Final Considerations

Discovery of new bioactive natural products isolated from marine sponges is a complex and multidisciplinary endeavor, where no traditional empirical knowledge and ethnopharmacology research exists to support this zoochemical exploration. Therefore, it is essential construct a solid database to fill this gap in the scientific literature on natural products discovery.

The present review describes the research on marine sponge derived cytotoxic compounds carried out between 2010 and 2012. This overview resulted in more than 73 articles closely correlated, attesting for instance, the great chemodiversity of these organisms as source of natural products. During this triennial database search, 337 secondary metabolites were isolated of which more than 197 ones were reported as novel chemical structures. Several of these new compounds, and their synthetic analogs, have shown confirmed in vitro cytotoxic and pro-apoptotic activities against various tumor/cancer cell lines bioassays. Moreover, some of them are of current interest for further in vivo evaluation.

The major structural classes of these new natural products were limited, in particular, to the terpenoids (41.9%), alkaloids (26.2%), macrolides (8.9%) and peptides (6.3%), which represented the four main chemical classes of compounds discovered from sponges in this period, which together with other chemical classes as polyketides, sterols, and others (16.7%) showed a range of biological activities.

Dictyoceratida, Haplosclerida, Tetractinellida, Poecilosclerida, and Agelasida were the key orders of sponges studied during this period. The examination of the contribution from an individual species revealed that regardless of the order, each species contributed, on average, 2–5 new compounds. The high number of new compounds was the result of the high diversity of species from these particular orders. These results were similar to those compiled by Mehbub et al. [8], covering new bioactive compounds derived from marine sponges prospected between 2001 and 2010. The order Dictyoceratida was found to be the most prolific producer of new compounds among all the sponge orders studied. *Petrosia* and *Haliclona* (Haplosclerida), *Rhabdastrella* (Tetractinellida), and *Coscinoderma* and *Hyppospongia* (Dictyioceratida), were found to be the most promising genera because of their capacity for producing new bioactive compounds.

Cytotoxicity assays are mandatory to access the cytotoxic level of a new compound. In this review, cytotoxicity assays of the active compounds isolated from marine sponges were established through those most common anticancer drug screen panels which consisted in either in human [(CCRF-CEM, LH-60 promyelocytic; K-562 chronic myelogenous; CLL lymphocytic; UT-7; and THP-1 monocytic) leukemia; A-549 and H-460 lung, HeLa cervix; HCT-8 ileum; HCT-116 and SW-480 colon and HT-29 colorectal; SF-268 central nervous system, MALME-3, MDA-MB-231, and (LOX-IMVI amelanotic) melanoma; SN12C renal, PC-3 and DU-145 prostate; MCF-7 breast; KB nasopharyngeal epidermal and FaDu pharynx; BGC-823, SGC, and MKN-45 gastric; TSU-Pr1 series and 5637 superficial bladder, A-431 epidermoid, A-549 alveolar basal epithelial, QGY-77003 hepatoma, for example)] or murine (P388 and L1210 lymphocytic leukemias; B16 melanoma, JB6 Cl41 epidermal, and L5187Y lymphoma) cultured tumor cell-lines. However, in a few reports cytotoxic studies were very extensive and have included the NCI-60 [165] or JFCR-39 [166] human cancer cell line panels for in vitro anticancer drug screen. Both screening systems—against different cancer cell lines—are well-known platforms and powerful tools for identifying the relationship between anticancer drugs and cell lines (=cytotoxicity), for the discovery of molecular-targeted anticancer agents, and facilitate the selection of many compounds for active development as anticancer drug candidates.

Despite the fact that the distribution of cytotoxic activities versus taxonomic sources for these compounds has not been plotted graphically, a quantitative analysis presumes a reputable screening of novel natural products. However, this review has not resulted in a corresponding increase in the number of new drug candidates, once that nearly 2/3 of the observed bioactives (65%) were shown to be effective only at moderate or high micromolar concentrations (≥10 µM), which resulted in little interest by the researchers as potential active drugs. Then, to the best of our knowledge, potent leading compounds were not so expressive and, indeed; reconfirm the traditional bottleneck existent through the development cycle of these potential drugs.

Although it was not the aim of this revision to address preclinical and clinical aspects of the bioactives isolated, it is evident the existing gap in relation to the natural availability of this pharmacologically important bioactives to perform assays of this nature since they require increasing amounts of these substances. In this sense, the synthetic chemical purpose represents an important route of choice for manufacture preclinical drug candidates. Chemical synthesis shows, moreover, potential to confirm or revise chemical structures obtained from discovered natural products, as well as to provide novel analogues.

Sponge aquaculture and recombinant microorganisms engineered are becoming very attractive to support bioactives. An efficient production of sponge derived products is a promising strategy that deserves further attention in future investigations in order to address the limitations regarding sustainable supply of marine drugs. Surely, the establishment of a matrix renewable and diverse of production of bioactives will compensate the extremely scarcity of natural supply of cytotoxic agents, contributing for a sustainable production of these drugs.

Finally, to conclude, it is expected that this review can highlight the general importance of the cytotoxic factors involved in the accurate and selective process used to discovery and development of new functional anticancer drugs.

## Figures and Tables

**Table 1:** Marine sponge families less explored about for new chemical features and their cytotoxic effects on different cancer cell lines.

Species (Order, Family)	Compounds	Cancer Cell Line (In Vitro Cytotoxicity)	Reference
*Biemna* sp. (Biemnida, Biemnidae)	Two pyridoacridines and the known isocystodamine **	K562 (ED_50_ = 5 nM for each compound)	[155]
*Cinachyrella enigmatica* (Tetractinellida, Tetillidae)	Enigmazole-A *	NCL–H60 (mean GI_50_ of 1.7 µM)	[156]
*Clathria* sp. (Poecilosclerida, Microcionidae)	Mirabilins H–J and three known mirabilins	ECACC, AGS, HT29 and int-407 (LD_50_ values > 30 µM)	[157]
*Haliclona* sp. (Haplosclerida, Chalinidae)	Eight cyclic bis-1,3-dialkylpyridiniums and two known cyclostellettamines	A549 (LC_50_ = 14.7–28.9 µM)	[158]
*Hyatella* sp. (Dictyoceratida, Spongiidae)	Five new scalarane sesterterpenes and six known compounds	K562 (LC_50_ = 14.8–39.5 µM); one compound with LC_50_ > 100 µM	[159]
*Myrmekioderma dendyi* (Axinellida, Heteroxyidae)	Myrmekioside E, and peracetylated myrmekioside E (myrmekioside E-2)	NSCLC-N6 (IC_50_ = 7.3 µM); A549 (IC_50_ = 9.7 µM)	[160]
*Spirastrella abata* (Clionaida, Spirastrellidae)	Three phingosine 4-sulfates, and lysophosphatidylglycerol	K562 (LC_50_ = 4–8 µM)	[161]
*Theonella swinhoei* (Tetractinellida, Theonellidae)	Swinholide J, and the known swinholide A	KB (IC_50_ = 6.0 nM)	[162]

* Compound has been synthesized, according to Skepper et al. [163]; ** Isocystodamine has been synthesized, according to Yoshiyasu et al. [164].
